# LncRNA SNHG8 regulates the migration and angiogenesis of pHUVECs induced by high glucose via the TRPM7/ERK_1/2_ signaling axis

**DOI:** 10.1038/s41598-023-49779-7

**Published:** 2023-12-18

**Authors:** Zongcheng Fan, Xin Chen, Laicheng Wang, Jianjian Yu, Shunpeng Zhang, Changsheng Xu, Jinxiu Lin, Yunchai Lin, Feng Peng

**Affiliations:** 1 The First Clinical Medical College, Fujian Medical University, Fuzhou, Fujian China; 2 Department of Cardiology, The Third People’s Hospital of Hefei, Hefei, Anhui China; 3 Department of Cardiology, National Regional Medical Center of Binhai Campus of the First Affiliated Hospital of Fujian Medical University, The First Affiliated Hospital of Fujian Medical University, Fuzhou, Fujian China; 4 Fujian Provincial Institute of Hypertension, The First Affiliated Hospital of Fujian Medical University, Fuzhou, Fujian China

**Keywords:** Cell biology, Molecular biology, Cardiology, Medical research, Molecular medicine

## Abstract

This study aimed to evaluate the regulatory effect and molecular mechanism of long noncoding RNA small nucleolus RNA host gene 8 (LncRNA SNHG8) in the migration and angiogenesis of primary human umbilical vein endothelial cells (pHUVECs) under high-glucose (HG) conditions. The HG-induced endothelial injury model was established in vitro.The cell model of silencing SNHG8, overexpressing SNHG8, and silencing TRPM7 was established by transfecting SNHG8-siRNA, SNHG8 plasmid and TRPM7-siRNA into cells with liposomes.The SNHG8 level was determined through reverse transcription-quantitative polymerase chain reaction (RT-qPCR). The expression levels of transient receptor potential melastatin 7 (TRPM7), endothelial nitric oxide synthase (eNOS), p-eNOS, extracellular signal-regulated kinase 1/2(ERK1/2), and p-ERK1/2 were assessed through western blot. Nitric oxide (NO) levels were measured with DAF-FM. pHUVEC migration was examined through wound healing and Transwell assay, and pHUVEC angiogenesis was observed through a tube formation assay. Results showed that HG promoted the expression of lncRNA SNHG8 and TRPM7 and decreased the ratio of p-eNOS/eNOS and p-ERK_1/2_/ERK_1/2_ in pHUVECs . NO production, migration , and angiogenesis were inhibited in pHUVECs under HG conditions. Silencing lncRNA SNHG8 and TRPM7 could significantly reverse the HG-induced decrease in eNOS activation, NO production , migration, and angiogenesis . SNHG8 and U0126 (ERK pathway inhibitor) overexpression enhanced the HG effects, whereas using U0126 did not affect the TRPM7 expression. In conclusion, lncRNA SNHG8 participates in HG-induced endothelial cell injury and likely regulates NO production, migration, and angiogenesis of pHUVECs via the TRPM7/ERK_1/2_ signaling axis.

## Introduction

Diabetes mellitus (DM) is one of the major global public health challenges^1^. Diabetic vascular disease is the leading cause of disability and premature mortality for patients with DM^2^. Injury and dysfunction of vascular endothelial cells (ECs), which are directly exposed to high glucose (HG), are major initiators of diabetic vascular disease^3^. Diabetic vascular disease exhibits impaired endothelium-dependent vasomotor effects and abnormal survival, growth, and migration of ECs; among them, migration capability is critical for angiogenesis^4^. Therefore, endothelial dysfunction induced by hyperglycemia should be improved for the prevention and treatment of diabetic vascular disease.

Extracellular signal-regulated kinase 1/2 (ERK1/2) is a key mitogen-activated protein kinase. After being phosphorylated by various growth factors, hormones, and cytokines, ERK_1/2_ can participate in meiosis, mitosis, and other functions, regulating cell growth, proliferation, differentiation, migration, and survival^5,6^. Nitric oxide (NO) is one of the proliferation-associated antiatherogenic vasoprotective factors. Phosphorylated endothelial nitric oxide synthase (eNOS) is the major source of NO production in ECs. ERK_1/2_ can induce NO production in ECs via eNOS activation^7^ and promote the proliferation, migration, and angiogenesis of human umbilical vein endothelial cells (HUVECs)^8^. Transient receptor potential melastatin 7 (TRPM7) is a membrane protein with kinase and ion channel function; when activated by HG, shear stress, oxidative stress, and other stimulations, it can impair EC function by inhibiting the ERK1/2 pathway^9,10^^.^ Silencing TRPM7 can increase phosphorylated ERK1/2, upregulate the expression of eNOS and NO, and promote endothelium-dependent vasomotor effects^11^.In addition, TRPM7 protein level was increased in high-glucose treated cells and diabetic mice, which reduced NOS level and increased apoptosis, inducing cell damage^12^.

Long noncoding RNAs (LncRNAs) are RNA transcripts with a length of over 200 nucleotide units without protein-coding functions^13^; they participate in the initiation and development of atherosclerosis^14^^.^ Small nucleolus RNA host gene 8 (SNHG8), a novel small nucleolar RNA located at 4q26, is involved in multiple biological functions, such as translation, transcription, RNA splicing, transcription regulation, and signal transduction^15^. Tao et al. demonstrated that SNHG8 promotes the proliferation, migration, invasion, and metastasis of gastric cancer, and the upregulation of SNHG8 expression in gastric cancer morbidity increases^16^. SNHG8 can inhibit human islet β-cell proliferation, reduce insulin levels, and increase blood glucose levels through nucleosome assembly protein 1-like 1 (NAP1L1) to inhibit the transcription of p57 (Kip2)^17,18^. Our previous FIESTA study also suggested that lncRNA SNHG8 is highly expressed in patients with incrased fasting glucose^19^. Huang et al. showed that lncRNA SNHG8 interacts with Epstein–Barr virus proteins LF3, BHLF1, BHRF1, and BNLF2a through co-expression and enrichment analysis. These proteins in turn regulate the expression of TRPM7, TRIM28, EIF4A2, NAP1L1, PLD3, and RPL18A^16^. It suggests an indirect regulatory relationship between lncRNA SNHG8 and TRPM7. However, the role of the SNHG8/TRPM7 pathway in diabetic vascular EC dysfunction remains unknown.

On the basis of these findings, we conducted this study to investigate the effects of lncRNA SNHG8 on the NO production, migration, and angiogenesis of HG-induced HUVECs and determine whether lncRNA SNHG8 could promote TRPM7 expression and then inhibit ERK1/2 pathway activation to cause endothelial dysfunction. These results provided a theoretical basis for screening new antidiabetic angiopathy drug targets.

## Methods and materials

### Cell culture and identification

Primary human umbilical vein endothelial cells (pHUVECs) were isolated from a neonatal umbilical cord through type I collagenase digestion method^20^. They were cultured in ECM (Cat. no. 001; ScienCell, USA) medium containing 5% FBS, 0.1% ECG, 100 U/mL penicillin, and 100 μg/mL streptomycin and incubated in 5% CO_2_ at 37 °C. The ECs presented a paving stone-like pattern under a microscope. Immunofluorescence Factor VIII (Cat. no. AM-0022; ZSGB, China) and CD31 (Cat. no. A4900; ABclonal, China) antibodies for identification were positive in pHUVECs.Cell identification results are shown in supplementary material. Studies were only performed with cells at passages 3–6. The study was approved by The Animal Medical Ethics Committee of Fujian Medical University (research license number: FJMU IACUC 2022-NSFC-0376). Informed signed consent was obtained from all subjects involved in the study. All experiments and methods were performed in accordance with relevant guidelines and regulations.

### Cell transfection

An SNHG8-expressing plasmid (pCDNA3.1 vector) and siRNAs targeting SNHG8 (si-SNHG8), TRPM7 (si-TRPM7), negative control (si-NC) were assembled by Hanheng Biotechnology (Shanghai, China). pHUVECs were transfected using LipoFiter 3.0 (Hanheng, China) in accordance with the manufacturer’s instructions. The cells were treated with 30 mM D-glucose (Cat. no. G7021; Sigma, USA) for 72 h and 10^–5^ M U0126 (Cat. no. S1901; Beyotime, China)^21^ for 30 min.

### Reverse transcription-quantitative PCR (RT-qPCR)

According to the manufacturer’s instructions, RNA was extracted with TRIZOL reagent (Cat. no. ER501-01; Transgen, China) and reversely transcribed into cDNA templates by using a reverse transcription kit (Cat. no. 11141ES60; Yeasen, China). SYBR Green master mix (Cat. no. 11202ES03; Yeasen, China) was used for qPCR amplification. Relative quantification was performed using the 2^−ΔΔCt^ method and normalized to β-actin^22^^.^ The following primer sequences were used: β-actin, forward 5ʹ-GCATGGAGTCCTGTCGCATCC-3ʹ and reverse 5ʹ-GCGGCCAGGATGGAGCCGC-3ʹ; SNHG8, forward 5ʹ-GCTGAGCTGAACACATTACG-3ʹ and reverse 5ʹ-ACGATAAGTCCATTGCCGGA-3ʹ.

### Western blot analysis

Western blot was performed as previously reported^23^. The total protein from the cells was extracted using RIPA lysed buffer (Cat. no. P0013B; Beyotime, China), and its quality was determined using a BCA kit (Cat. no. P0012S; Beyotime, China). Proteins were loaded onto 4%–20% SDS-PAGE (Cat. no. F11420Gel; ACE, China) and transferred to PVDF membranes (Cat. no. IPVH00010; Merck Millipore, Germany). According to the molecular weight of the protein, the reference and target proteins were cut separately at the corresponding positions of the membrane.The membranes were then exposed to 5% skim milk and probed with primary antibodies against TRPM7 (1:1000; ab262698; Abcam), eNOS (1:1000; abB252439; Abcam), phosphorylation-eNOS (1:1000; ab215717; Abcam), ERK1/2 (1:1000; ab184699; Abcam), phosphorylation-ERK1/2 (1:1000; ab278538; Abcam), and β-actin antibody (1:10,000; AC026; ABclonal) at 4 °C overnight. They were incubated with secondary antibodies HRP-goat anti-rabbit (1:10,000; AS014; ABclonal) at room temperature for 1 h. Protein bands were detected on a chemiluminescence system by using an ECL kit (Cat. no. P10100; NCM, China).

### NO measurements

After cell transfection and high glucose treatment, each six-well plate was added with 1 ml of DAF-FM DA (dilution 1:1000; Beyotime, China) with a final concentration of 5 μmol/l. The sample was incubated for 20 min and washed thrice with phosphate-buffered saline (PBS). Cells were randomly selected for visualization and photographed under a fluorescence microscope Objective lens  100× (Olympus, Japan). Immunofluorescence staining was quantitatively assessed with ImageJ 1.6.

### Wound healing assay

After cell transfection and high glucose treatment, pHUVECs were planted equally into a six-well plate and cultured to 80–90% confluent. Then, the cells were incubated in the medium with 1% FBS, and vertical scratches were drawn by a 200 μL pipette tip. The cells were photographed by Inverted microscopy OLYMPUS DP71/72(Objective lens  100×) at 0, 24 h after scraping. Wound healing percentage was used to evaluate migratory abilities of pHUVECs. Wound healing percentage = [ (A – B)/A] × 100%, where A is the average wound surface area at 0 h and B is the average wound surface area at 24 h. Wound surface area was measured by Image J.

### Cell migration assay

Transwell chambers (Cat. no. 3422; Corning, USA) were used for cell migration assays. In this procedure, 200 μl of pHUVEC suspension at a density of 1 × 10^5^/chamber were added to the serum-free medium in the upper chamber, and the relevant medium containing 10% FBS was added to the lower chamber as a chemoattractant. After being incubated for 24 h, the cells in the upper chamber were removed using cotton swabs, while the cells in the lower chamber were fixed with formaldehyde and stained with 0.1% crystal violet staining solution (Cat. no. C0121; Beyotime, China) for 20 min. They were then imaged under a microscope(Objective lens  200×). The number of migrated cells was calculated using ImageJ.

Tube formation assay. Matrigel (Cat. no. 356234; Corning, USA), 48-well culture plates, and pipette tips were exposed to 4 °C overnight. After being precooled, the Matrigel was spread in 48-well plates and cured in incubators at 37 °C for 30 min. Then, 200 μl of pHUVEC suspension (1.5 × 105 PCS/ml) was added to the Matrigel surface and incubated at 37 °C for 4–6 h. Then, the Matrigel was imaged under a microscope(Objective lens  100×) to quantify tube formation for each plate and analyzed using ImageJ.

### Statistical analysis

The procedures were repeated thrice. Quantitative data were evaluated using GraphPad Prism 8 (GraphPad Software Inc., San Diego, CA, USA). All experimental data were quantitative data and presented as means ± SEs. Student’s t-test was conducted to compare the difference between two groups, and one-way ANOVA was conducted for multiple groups. Pairwise comparisons between groups were applied through the least significant difference (LSD) method. Results were statistically significant when P < 0.05.

## Results

### HG promoted the lncRNA SNHG8 expression in pHUVECs

**Figure 1:**
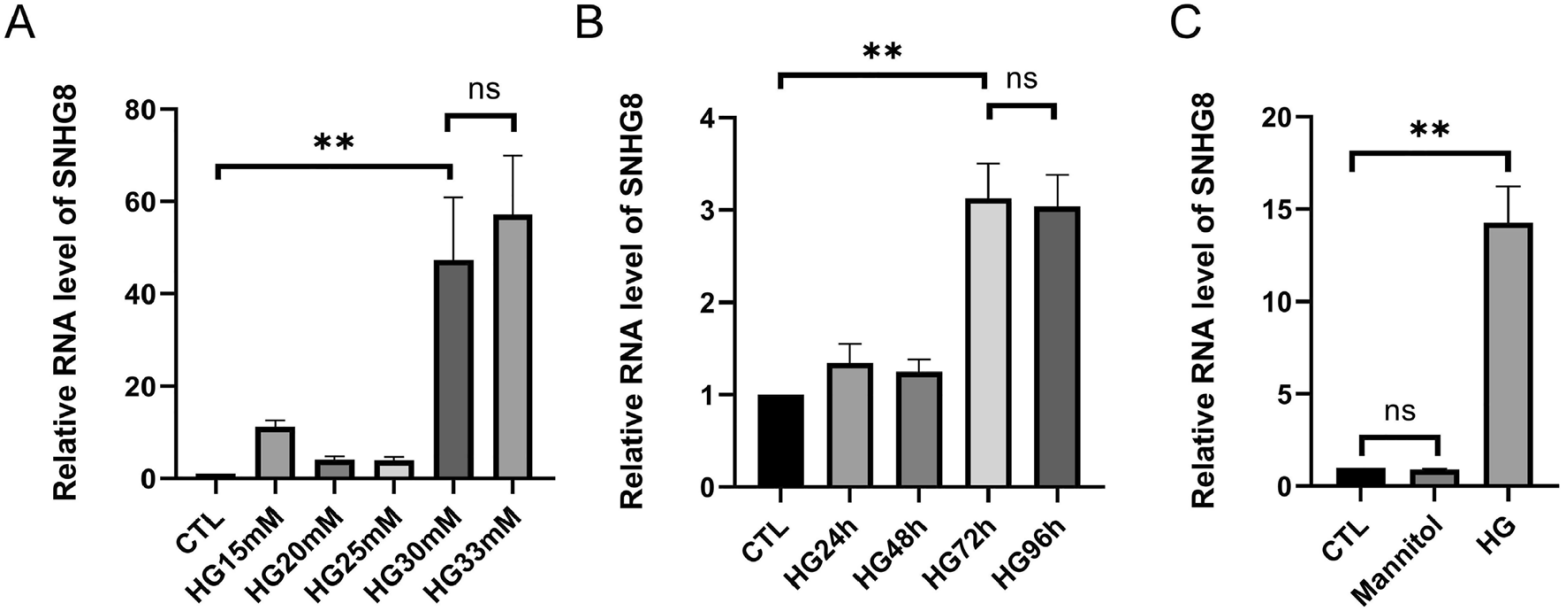
HG treatment promotes lncRNA SNHG8 expression in pHUVECs. (**A**) pHUVECs were stimulated with 5.5 mM glucose (CTL), 15 mM, 20 mM, 25 mM and 33 mM glucose (HG) for 72 h. (**B**) pHUVECs were stimulated with HG (30 mM) for 24, 48, 72, and 96 h. (**C**) pHUVECs were stimulated with HG (30 mM) and Mannitol (30 mM) for 72 h. The level of lncRNA SNHG8 was detected by RT-qPCR. (**, P < 0.01; ns, P > 0.05; n = 3).

To investigate the effect of SNHG8 on ECs under HG condition, we detected the SNHG8 expression after the cells were exposed to 5.5 mM glucose (CTL group) to 33 mM glucose for 72 h and to 30 mM glucose for 0 h (CTL group) to 96 h. We used mannitol as an osmolality control. We found that as the glucose concentration increased, the SNHG8 expression showed a concentration-dependent induction of expression (P < 0.01, Fig. 1A). SNHG8 did not differ between HG 30 mM and HG 33 mM groups (P > 0.05, Fig. 1A).HG 72 and 96 h groups significantly enhanced the SNHG8 expression (P < 0.01, Fig. 1B). SNHG8 did not differ between HG72 h and HG96 h groups, and CTL and mannitol groups, (P > 0.05, Fig. 1B, C).

### HG downregulated the eNOS activation and NO production of pHUVECs via SNHG8

**Figure 2:**
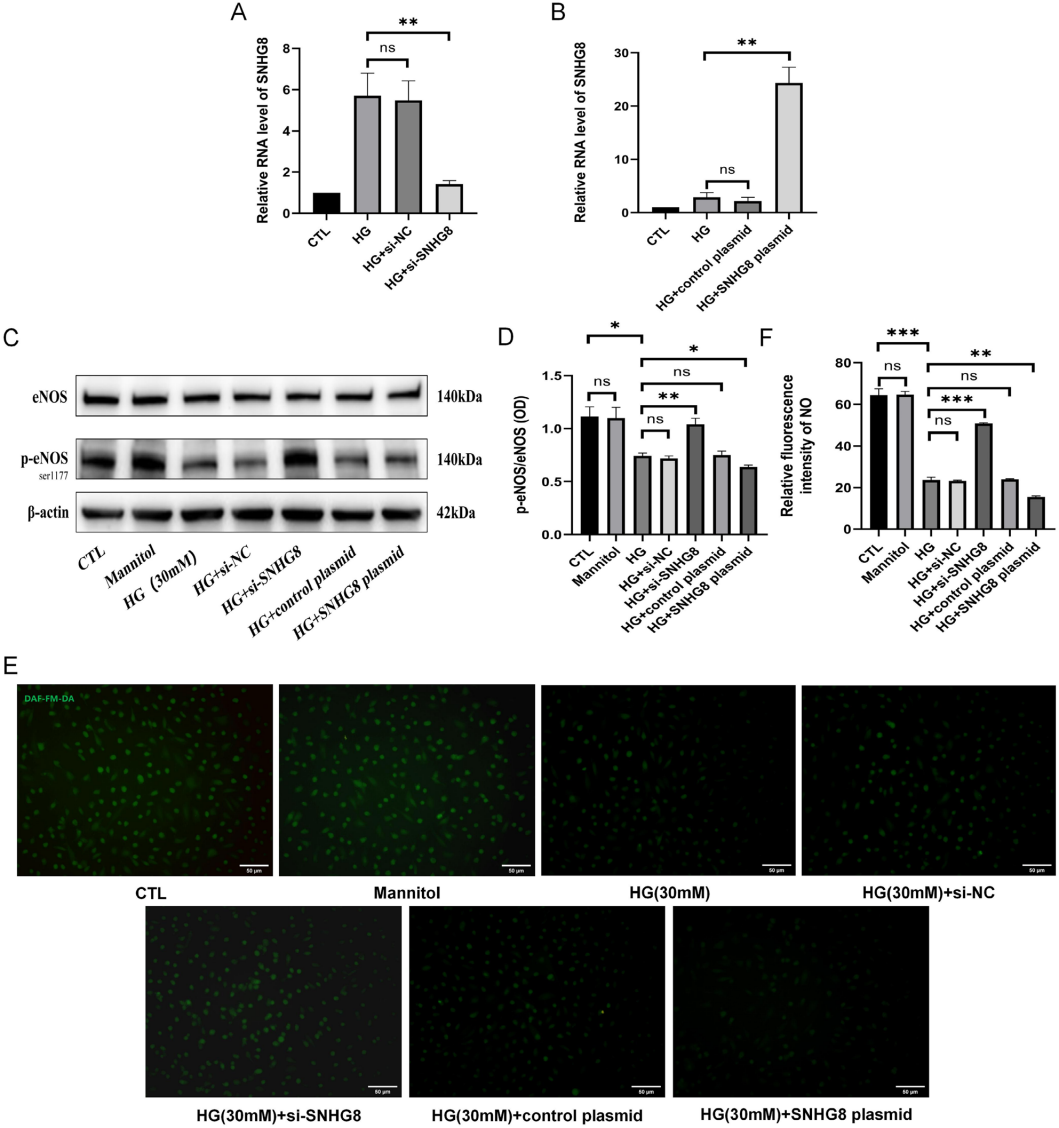
LncRNA SNHG8 regulated eNOS activity and NO production in HG-stimulated pHUVECs. pHUVECs were transfected with SNHG8 siRNA and SNHG8 pcDNA3.1 plasmid by LipoFiter3.0 transfection for 6 h; the culture medium was changed (negative control group was set) and stimulated with HG (30 mM) for 72 h. (**A**) siRNA-SNHG8 was transfected into pHUVECs. (**B**) SNHG8 plasmids were transfected into pHUVECs. (**C**) Western blot analysis of the eNOS and p-eNOS expression levels in pHUVECs. (**D**) Quantitative analysis of eNOS activity, denoted by p-eNOS/eNOS (OD) ratio. (**E**) DAF-FM-DA fluorescent probe was used to detect NO production in pHUVECs (scale = 50 μm). (**F**) Quantitative analysis of NO production, denoted by green fluorescence intensity. The level of lncRNA SNHG8 was detected through RT-qPCR (*, P < 0.05; **, P < 0.01; ***, P < 0.001, ns, P > 0.05; n = 3).

The cells were transfected with siRNA against SNHG8, scrambled siRNA (siRNA NC), or lipofectamine alone and cultured with 30 mM glucose for 72 h. The expression efficiency of SNHG8 was detected through RT-qPCR. The results showed that the SNHG8 expression in the HG + si-SNHG8 group was significantly lower than that in the HG group, and the transfection efficiency was > 70% (P < 0.01, Fig. 2A). No significant difference was found between HG and HG + Si-NC groups (P > 0.05, Fig. 2A). The cells were transfected with SNHG8 plasmid, Empty plasmid vector(CTL plasmid), and cultured with 30 mM glucose for 72 h. The expression efficiency of SNHG8 was detected through RT-qPCR. The results showed that the SNHG8 expression in the HG + SNHG8 plasmid group was significantly higher than that in the HG group, and the overexpression efficiency was > 300% (P < 0.01, Fig. 2B). No significant difference was found between HG and HG + control plasmid groups (P > 0.05, Fig. 2B).

Furthermore, we conducted Western blot to evaluate eNOS and p-eNOS expression and used the fluorescent probe DAF-FM DA to assay the NO production. Western blot and DAF-FM DA assay showed that HG significantly reduced the ratio between p-eNOS and total eNOS (P < 0.05, Fig. 2C, D) and downregulated NO production (P < 0.001, Fig. 2E, F). SNHG8 further reduced p-eNOS and NO production (P < 0.01, Fig. 2C–F).Si-SNHG8 significantly reversed these trends (P < 0.05 and P < 0.001, Fig. 2C–F). The p-eNOS/eNOS ratio and NO production did not significantly differ between the CTL and mannitol groups, the HG and HG + si-NC groups, and the HG and HG + control plasmid groups (P > 0.05, Fig. 2C–F). Therefore, HG stimulated LncRNA SNHG8 to regulate the eNOS activation and NO production of pHUVECs.

### SNHG8 regulated the migration and angiogenesis of HG-induced pHUVECs

**Figure 3:**
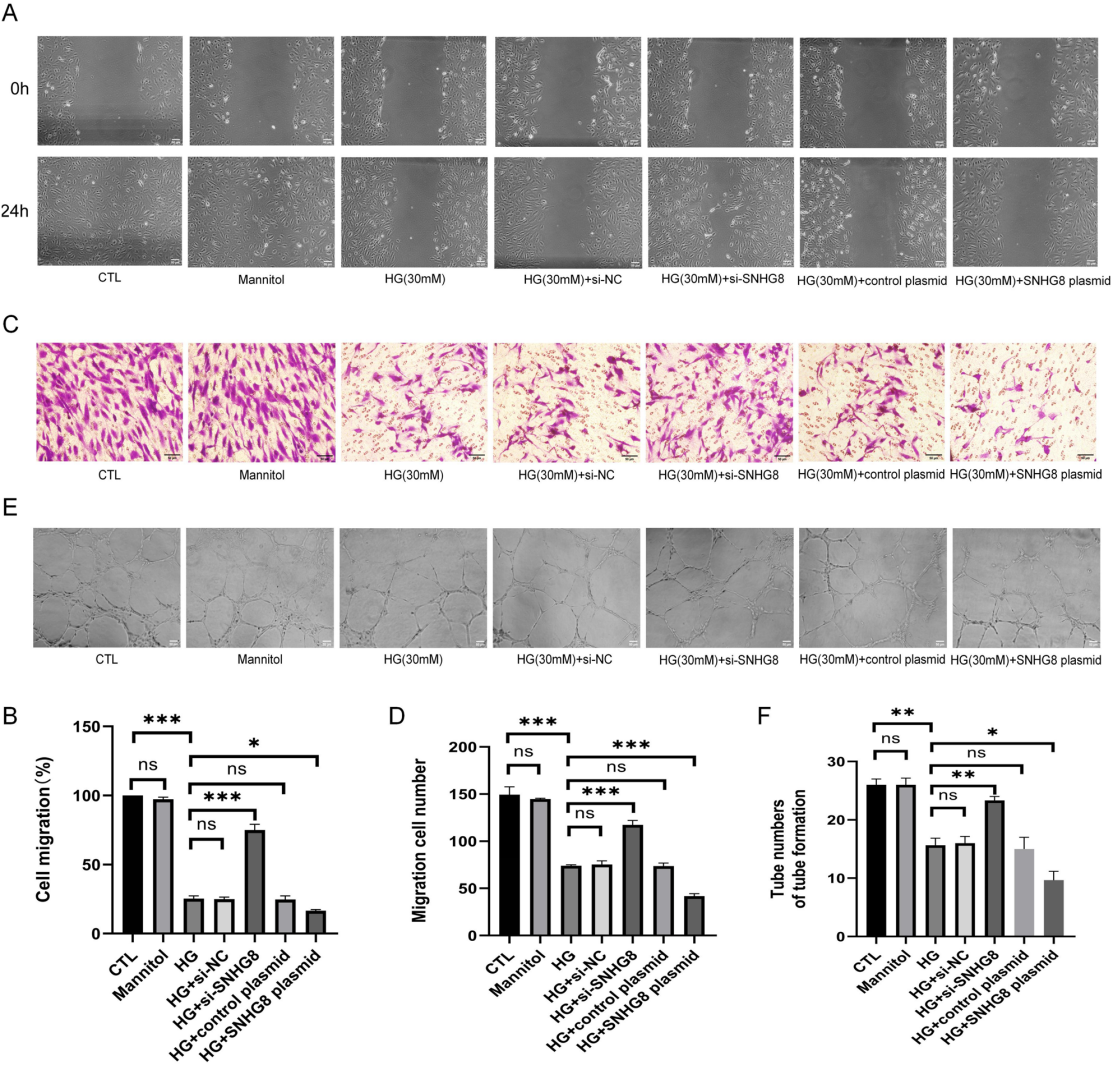
LncRNA SNHG8 regulated the migration and angiogenesis of pHUVECs under HG stimulation. pHUVECs were transfected with SNHG8 siRNA and SNHG8 pcDNA3.1 plasmid through LipoFiter3.0 transfection for 6 h. The culture medium was changed (negative control group was set up) and stimulated with HG (30 mM) for 72 h. (**A**) pHUVEC migration was detected through a scratch test (scale = 50 μm). (**B**) Quantitative analysis of the scratch test; the migration level of pHUVECs was expressed as the healing rate. (**C**) pHUVEC migration was detected through a Transwell assay (scale = 50 μm). (**D**) Quantitative analysis of the Transwell assay by using the number of the transplanted cells to represent the migration level of pHUVECs. (**E**) pHUVEC angiogenesis was detected using a tube formation test (scale = 50 μm). (**F**) Quantitative analysis of the tube formation test; the number of tubes of pHUVECs as the angiogenesis level expression (*, P < 0.05; **, P < 0.01; ***, P < 0.001, ns, P > 0.05; N = 3).

In Fig. 3, wound healing and Transwell assay were used to evaluate the effect of the migratory abilities of pHUVECs, and the tube formation assay on Matrigel were used to assess the angiogenic capability. The migratory ability (P < 0.001, Fig. 3A–D) and angiogenic ability (P < 0.01, Fig. 3E, F) of pHUVECs were reduced under HG conditions compared to control group. SNHG8 siRNA significantly reversed these trends (P < 0.001, Fig. 3A–D and P < 0.01, Fig. 3E, F). Migration and angiogenesis could be further suppressed by SNHG8 plasmid (P < 0.05, Fig. 3A–F). The migratory and angiogenic capability did not significantly differ between the CTL and mannitol groups, the HG and HG + si-NC groups, and the HG and HG + control plasmid groups (P > 0.05, Fig. 3A–F). Therefore, lncRNA SNHG8 could regulate the migration and angiogenesis of HG-induced pHUVECs.

### SNHG8 regulated the TRPM7/ERK1/2 pathway of pHUVECs under HG condition

**Figure 4:**
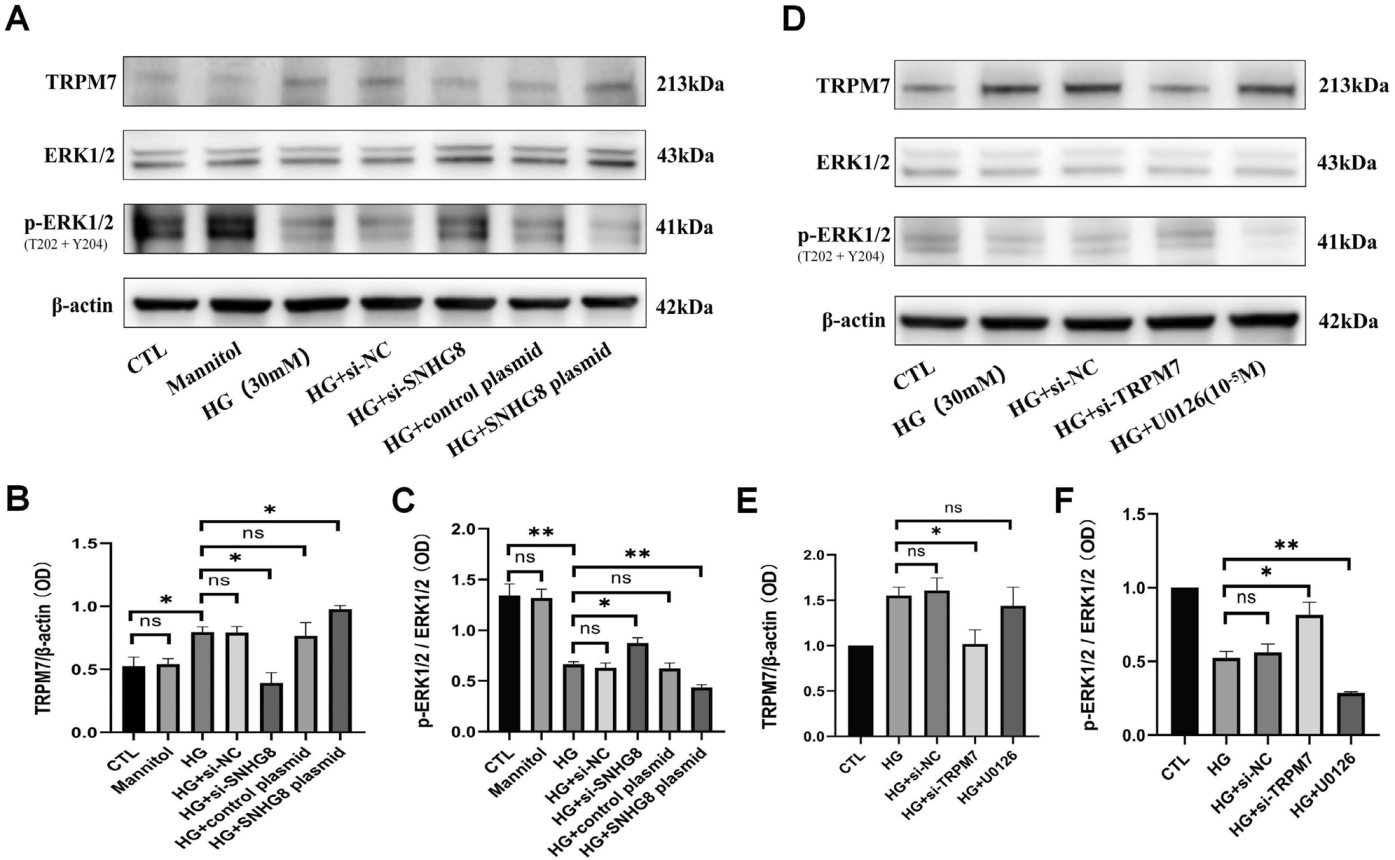
LncRNA SNHG8 regulated the TRPM7/ERK_1/2_ signaling pathway in HG-stimulated pHUVECs. pHUVECs were transfected with SNHG8 siRNA and SNHG8 pcDNA3.1 plasmid by LipoFiter3.0 for 6 h. The culture medium was changed (negative control group was set up) and stimulated with HG (30 mM) for 72 h. (**A**) Western blot analysis of TRPM7, ERK1/2, and p-ERK1/2 expression levels in pHUVECs. (**B**) Quantitative analysis of TRPM7 expression levels. (**C**) Quantitative analysis of ERK1/2 activity, denoted by the p-ERK_1/2_/ERK_1/2_ (OD) ratio. Subsequently, pHUVECs were transfected with TRPM7 siRNA by LipoFiter3.0 for 6 h. The culture medium was changed (negative control group was set up), and HG (30 mM) was added for 72 h,10^-5^ M U0126 (ERK pathway inhibitor) for 30 min. (**D**) Western blot analysis of TRPM7, ERK1/2, and p-ERK1/2 expression. (**E**) Quantitative analysis of TRPM7 expression. (**F**) Quantitative analysis of ERK1/2 activity, denoted by the p-ERK_1/2_/ERK_1/2_ (OD) ratio. (*, P < 0.05; **, P < 0.01; Ns, P > 0.05; N = 3).

**Figure 5:**
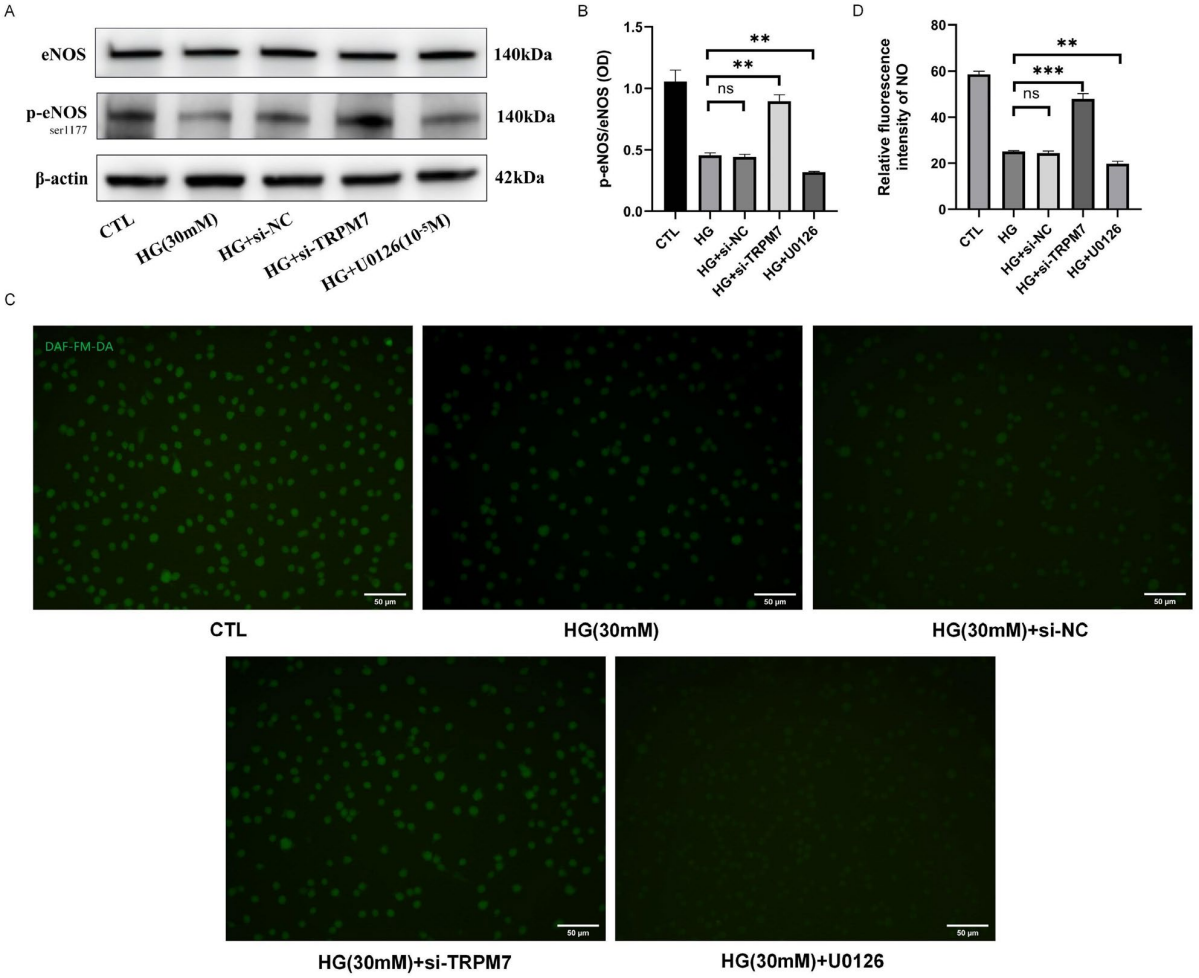
TRPM7/ERK_1/2_ signaling pathway regulated the eNOS activity and NO production of pHUVECs stimulated by HG. pHUVECs were transfected with SNHG8 siRNA and SNHG8 pcDNA3.1 plasmid by LipoFiter3.0 transfection for 6 h. The culture medium was changed (negative control group was set up) and stimulated with HG (30 mM) for 72 h. (**A**) Western blot analysis of eNOS and p-eNOS expression levels in pHUVECs. (**B**) Quantitative analysis of eNOS activity, denoted by p-eNOS/eNOS (OD) ratio. (**C**) DAF-FM-DA fluorescent probe was used to detect NO production in pHUVECs (scale = 50 μm). (**D**) Quantitative analysis of NO production, denoted by green fluorescence intensity. (**, P < 0.01; ***, P < 0.001, ns, P > 0.05; n = 3).

To investigate the possible regulatory relationship between the lncRNA SNHG8 and TRPM7/ERK_1/2_ pathway, we conducted Western blot to determine the expression of TRPM7, ERK_1/2_, p-ERK_1/2_ after SNHG8 was silenced and overexpressed. HG significantly increased the TRPM7 expression (P < 0.05, Fig. 4A, B) and decreased the ERK_1/2_ activation (P < 0.01, Fig. 4A, C). Si-SNHG8 significantly decreased the TRPM7 expression (P < 0.01, Fig. 5A, B) and increased the ERK_1/2_ activation (P < 0.01, Fig. 4A, C). While SNHG8 plasmid further increased the TRPM7 expression (P < 0.05, Fig. 4A, B) and decreased the ERK_1/2_ activation (P < 0.01, Fig. 4A, C). No significant differences in the TRPM7 expression and decreased ERK_1/2_ activation were found between the CTL and mannitol groups, the HG and HG + si-NC groups, and the HG and HG + control plasmid groups (P > 0.05, Fig. 4A–C).

To further confirm the regulatory relationship between TRPM7 and ERK_1/2_, we transfected si-TRPM7 through lipofection to silence TRPM7 and used U0126 (ERK pathway inhibitor) to inhibit ERK_1/2_ activation under HG conditions. Western blot results indicated that si-TRPM7 significantly increased ERK_1/2_ activation (P < 0.05, Fig. 4D, F). U0126 significantly reduced ERK_1/2_ activation, whereas no significant deviation was found in the TRPM7 expression (P > 0.05, Fig. 4D, E). TRPM7 expression and ERK_1/2_ activation did not significantly differ between the HG and HG + si-NC groups (P > 0.05, Fig. 4D–F). These results suggested that lncRNA SNHG8 involved in regulating the TRPM7 expression and ERK_1/2_ activation of HG-induced pHUVECs. TRPM7 is an upstream molecule that regulates ERK_1/2_ activation.

### TRPM7/ERK1/2 pathway regulated the eNOS activation and NO production of pHUVECs under HG condition

In Fig. 5, we conducted Western blot and fluorescent probe DAF-FM DA test after TRPM7 was silenced and ERK_1/2_ activation was inhibited. Si-TRPM7 significantly increased the p-eNOS/eNOS ratio and NO production under HG (P < 0.01, Fig. 5A, B; P < 0.001, Fig. 5C, D). U0126 significantly decreased the p-eNOS/eNOS ratio and NO production under HG (P < 0.01, Fig. 5A–D). The p-eNOS/eNOS ratio and NO production had no significant differences between the HG and HG + si-NC groups (P > 0.05, Fig. 5A–D). These results suggested that the TRPM7/ERK1/2 pathway involved in eNOS activation and NO production regulation of pHUVECs induced by HG. These results suggested that the TRPM7/ERK1/2 pathway involved in the eNOS activation and NO production regulation of pHUVECs induced by HG.

### TRPM7/ERK1/2 pathway regulated the migration and angiogenesis of pHUVECs under HG

**Figure 6:**
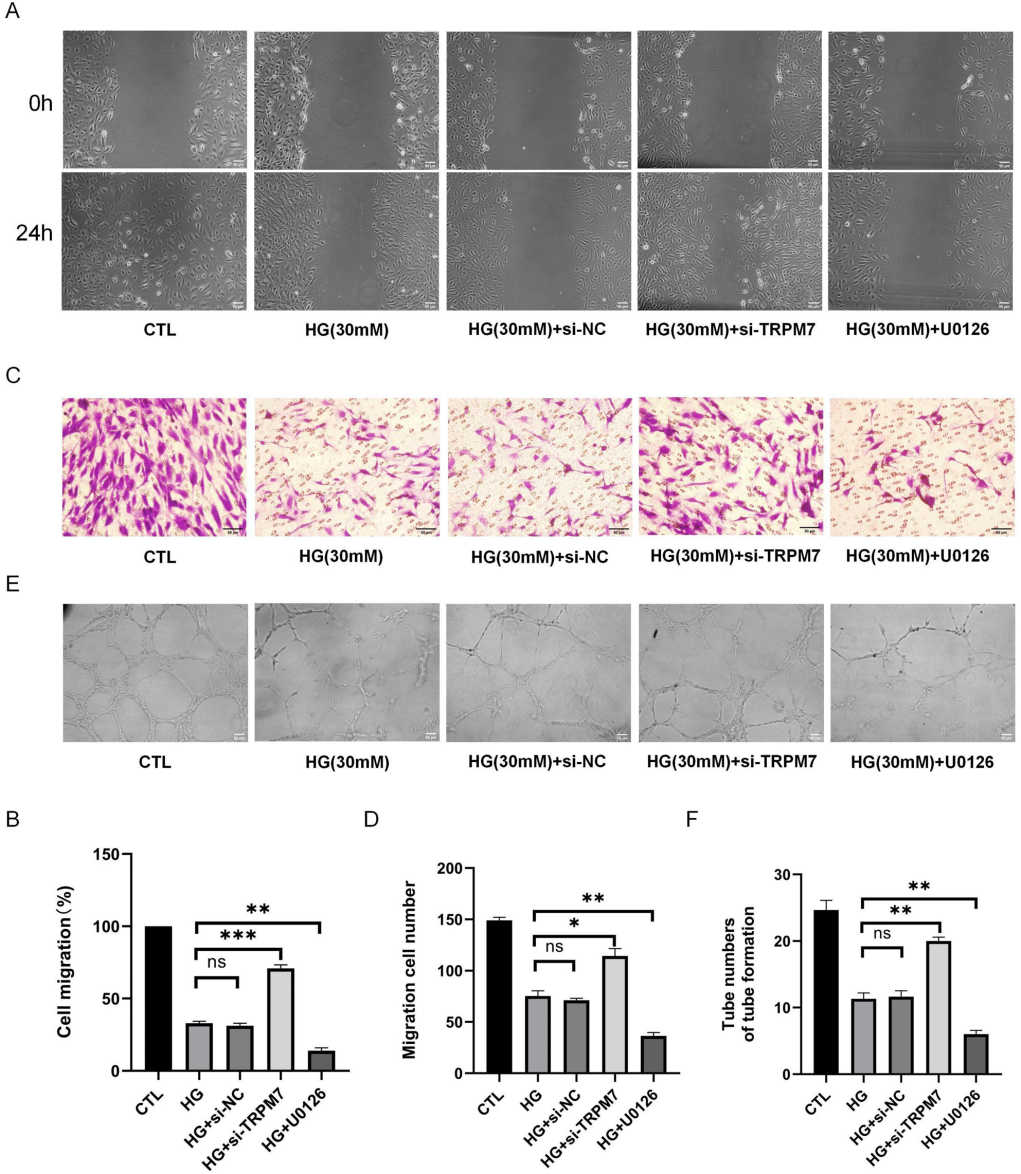
TRPM7/ERK_1/2_ signaling pathway regulates the migration and angiogenesis of pHUVECs under HG stimulation. pHUVECs were transfected with TRPM7 siRNA and treated with 10^–5^ M U0126 followed by HG (30 mM) stimulation for 72 h. (**A**) pHUVEC migration was detected via a scratch test (scale = 50 μm). (**B**) Quantitative analysis of the scratch test; the migration level of pHUVECs was expressed as the healing rate. (**C**) pHUVEC migration was detected via a Transwell assay (scale = 50 μm). (**D**) Quantitative analysis of the Transwell assay involving the number of transplanted cells to represent the migration level of pHUVECs. (**E**) Angiogenesis of pHUVECs was detected through a tube formation test (scale = 50 μm). (**F**) Quantitative analysis of the tube formation test; the number of tubes of pHUVECs as the expression of angiogenesis level (*, P < 0.05; **, P < 0.01; ***, P < 0.001, ns, P > 0.05; N = 3).

In Fig. 6, we conducted the scratch test, the Transwell assay, and the tube formation assay after TRPM7 was silenced and ERK_1/2_ activation was inhibited. The wound healing rate (P < 0.001, Fig. 6A, B), migration abilities (P < 0.05, Fig. 6C, D), and angiogenesis (P < 0.01, Fig. 6E, F) of the HG + Si-TRPM7 group significantly increased compared with that of the HG group. Conversely, U0126 significantly decreased the wound healing rate, migration abilities, and angiogenesis (P < 0.01, Fig. 6A–F). Migratory abilities and angiogenic capability had no significant differences between the HG and HG + si-NC groups (P > 0.05, Fig. 6A–F). These results suggested that the TRPM7/ERK_1/2_ pathway regulated the migration and angiogenesis of pHUVECs under HG condition.

## Discussion

The impairment of endothelium-dependent vessel relaxation and the dysregulation of angiogenesis are the main causes of vascular complications in diabetes^24^. Increased blood glucose levels induce the mitochondria to produce excessive ROS through the protein kinase C (PKC) pathway. Then, excessive ROS downregulates bioavailable NO levels by reducing the eNOS activity and leads to impaired vasorelaxation^25^. Angiogenesis plays an essential role in atherosclerosis^26^. Angiogenesis begins with vascular germination, and vascular endothelial growth factor (VEGF) binds to vascular endothelial growth factor receptor 2 (VEGFR2) expressed by ECs. VEGFR2 activation induces numerous signaling cascades that promote angiogenesis^27,28^. HG induces ECs to promote VEFGR2 phosphorylation and inhibit the binding to VEGF, thereby inhibiting angiogenesis^29^. In this study, pHUVECs were obtained from the umbilical cords of healthy newborns and used as model ECs. A pHUVEC HG-induced injury model was established, revealing that HG impaired the eNOS activity, NO production, migration, and angiogenesis ability of ECs. LncRNA SNHG8 was highly expressed under HG conditions. Moreover, silencing SNHG8 could retrieve HG-induced impairment of ECs, and SNHG8 overexpression could further stress the impairment. These results were consistent with the theory that HG promoted vascular endothelial vasodilatory and angiogenesis dysfunction.

LncRNAs are important gene expression and pathogenesis regulatory factors^30^, which serve as signals, bait, guide, or scaffold molecules by binding to proteins, DNAs, RNAs, and their combinations^31^. Former studies have mainly focused on their function as proliferation, migration, invasion promotor, and apoptosis inhibitor in tumors such as gastric cancer, breast cancer, and colorectal cancer^32^^.^ Yu et al. suggested that SNHG8 promotes breast cancer cell proliferation, migration, and invasion through the miR-634/ZBTB20 pathway^33^ . Likewise, Tian et al. indicated that SNHG8 participates in diffuse large B cell lymphoma cell proliferation and colony formation, thereby inhibiting cell apoptosis^34^. LncRNA SNHG8 also regulates non-neoplastic diseases. In a myocardial infarction model, SNHG8 can modulate the NF-kB signaling pathway to inhibit cell viability and induce apoptosis^35^. It can promote inflammatory reactions in the microvascular ECs of a mouse brain^36^. However, no functional studies about lncRNA SNHG8 in HUVECs impaired by HG have been performed. Our study suggested that SNHG8 was highly expressed in HG-induced pHUVECs, which was consistent with the results of our FIESTA study^19^. LncRNA SNHG8 initially increased, decreased, and increased further as the glucose concentration increased. Because no relevant articles on the SNHG8 expression under different glucose concentrations have been published, we cannot fully explain this phenomenon at present. A previous report in China showed that as the glucose concentration increased, cell proliferation and viability can be initially promoted and subsequently inhibited. This phenomenon may be caused by the effect of glucose on the energy metabolism, transcription, and secretion of cytokines of ECs, but it should be further investigated in the future. Furthermore, our results demonstrated that SNHG8 exerted inhibitory effects on migration and angiogenesis, which were different from tumor cell models. This heterogeneity relies on the characteristics of HG-induced pHUVECs, which are significantly different from cancer cells and the tumor microenvironment. Therefore, silencing SNHG8 expression could improve vascular endothelial function, which might provide a new strategy for preventing diabetic vascular disease.

TRPM7 is an important function molecule in HUVECs^37^. Its expression increases in HUVECs cultured with low serum magnesium levels, and silencing TRPM7 expression can promote HUVEC migration and angiogenesis^9^. Sun et al. suggested that downregulated TRPM7 expression in HUVECs stimulated by HG can decrease ROS generation and eNOS expression; it can also increase cell viability to improve HG-induced vascular EC damage^38^. TRPM7 is involved in the angiogenesis of ECs^39^. MiR-9-5p through the PI3K/AKT/autophagy pathway targeting TRPM7 to promote the proliferation, migration, and angiogenesis of ECs^40^.TRPM7 kinase is also a regulator of insulin synthesis, β-cell dynamics, and glucose homeostasis. TRPM7 reduces insulin secretion of β-cells by inhibiting AKT/ERK signaling pathway^41^. In addition, high glucose significantly increased NF-κB activity and ROS production in HUVECs^42^, and high glucose also promoted the upregulation of TRPM7 expression, which activated the transcription of NF-κB^43^. Recently, it has been found that high glucose induces the demethylation of NF-kB promoter region, which leads to the increase of its mRNA expression^44^.Therefore, the interaction between TRPM7 and NF-κB in high glucose conditions was not further explored in this study.

The Ras/Raf/MEK/ERK signaling pathway is closely correlated with the proliferation, migration, and angiogenesis of ECs. When Ras and GTP are combined in an activated state, Raf can be activated, and MEK1/2 can be further activated. Activated MEK1/2 releases ERK1/2 (T202/Y204), and activated ERK1/2 can be transferred to the nucleus and regulate cell proliferation, migration, and differentiation by regulating the transcription factor activity^45^^.^ For example, miR-155 downregulates ERK1/2 activation to reduce HUVEC damage and apoptosis and promotes HUVEC migration and capillary formation^46^. Silencing TRPM7 can promote ERK1/2 activation and myosin light chain (MLC) phosphorylation, attenuate HUVEC adhesion to the matrix, and promote cell migration, wound healing, and angiogenesis processes^10^^.^ In this study, TRPM7 was highly expressed, and the p-ERK1/2 expression was downregulated under the HG condition through Western blot assay. Furthermore, TRPM7 expression was downregulated by silencing lncRNA SNHG8 under HG, while p-ERK1/2 expression was upregulated, thereby improving the NO production, migration, and angiogenesis of pHUVECs; however, SNHG8 overexpression reversed this appeal. We found that silencing TRPM7 under HG condition could increase the ERK1/2 activity, improved the eNOS activity, migration, and angiogenesis ability of pHUVECs, while using U0126 (ERK pathway inhibitor) reversed these results, but did not affect the TRPM7 expression. These results suggested that the SNHG8/TRPM7/ERK_1/2_ signaling axis might regulate HG-induced EC injury.

## Conclusion

**Figure 7:**
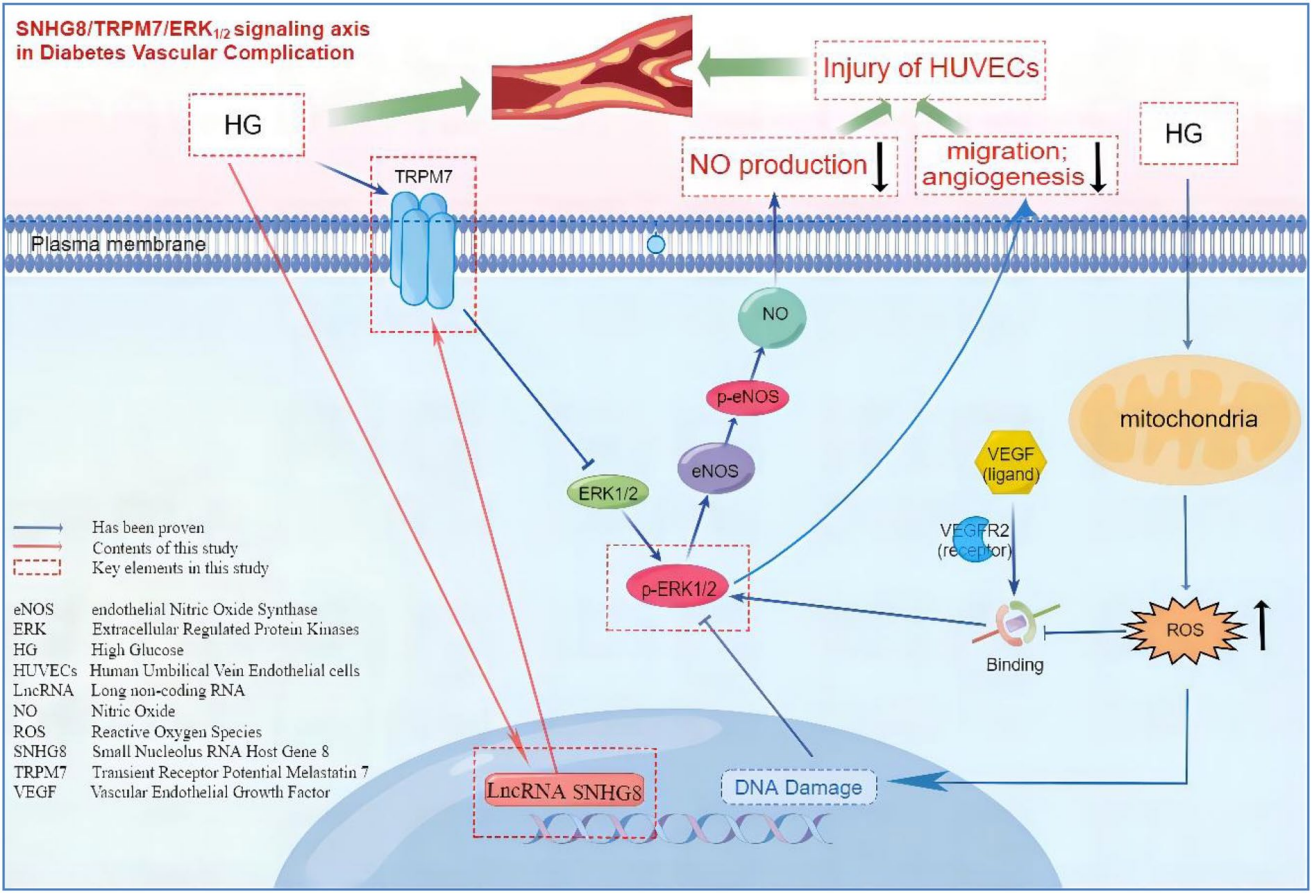
Schematic diagram representing the lncRNA SNHG8 plays an important role in HG-induced endothelial cell injury and may regulate the NO production, migration, and angiogenesis of pHUVECs via the TRPM7/ERK1/2 signaling axis.

LncRNA SNHG8 plays an important role in HG-induced endothelial cell injury and may regulate the NO production, migration, and angiogenesis of pHUVECs via the TRPM7/ERK_1/2_ signaling axis (Fig. 7). While previous studies on lncRNA SNHG8 mainly focused on cancer, this study is the first to investigate the regulatory role of SNHG8 in a HG-induced pHUVEC injury model and provide a theoretical basis for determining and screening new therapeutic targets for diabetic vascular disease.

### Supplementary Information

Supplementary Information.

## Data Availability

All the datasets generated and analyzed in the present study are available from the corresponding author on reasonable request.

## Abbreviations

LncRNA: Long non-coding RNA
SNHG8: Small nucleolus RNA host gene 8
TRPM7: Transient receptor potential melastatin 7
PHUVEC: Primary human umbilical vein endothelial cell
ERK1/2: Extracellular regulated protein kinases1/2
