# Supplementary material for: LncRNA SNHG8 regulates the migration and angiogenesis of pHUVECs induced by high glucose via the TRPM7/ERK1/2 signaling axis
Source: Sci Rep. 2023 Dec 18;13:22485. doi: 10.1038/s41598-023-49779-7 (PMC10728107; doi:10.1038/s41598-023-49779-7)
Supplement: Supplementary file 1 — Supplementary Information. [file 41598_2023_49779_MOESM1_ESM.pdf]

Supplementary information

Supplemental Tables

Supplemental Table 1. Antibodies used in the current study.

Target (Art.No.)	Application	Dilution	Vendor
CD31(A4900)	ICC	1:100	Abclonal(Wuhan,China)
Factor VIII(AM-0022)	ICC	1:100	ZSGB(Wuxi,China)
Goat IgG-FITC anti-mouse(AS001)	ICC	1:100	Abclonal
Goat IgG-FITC anti-rabbit(AS011)	ICC	1:100	Abclonal
β-actin(AC026)	Western blot	1:5000	Abclonal
TRPM7(ab262698)	Western blot	1:1000	Abcam(Cambridge, UK)
eNOS(ab252439)	Western blot	1:1000	Abcam
p-eNOS(ab215717)	Western blot	1:1000	Abcam
ERK1/2(ab184699)	Western blot	1:1000	Abcam
p-ERK1/2(ab278538)	Western blot	1:1000	Abcam
Goat IgG-HRP anti-rabbit(AS014)	Western blot	1:5000	Abclonal
Goat IgG-HRP anti-mouse(AS003)	Western blot	1:5000	Abclonal

Supplemental Table 2. SiRNA sequences used in this study

Oligo	Top strand (5'-3')	Bottom strand (5'-3')
hs-SNHG8-si-1	AGUUGUCUUUCGUAAGUUAdTdT	UAACUUACGAAAGACAACUdTdT
hs-SNHG8-si-2	GAUGGAUGAUGGAAACAUAdTdT	UAUGUUUCCAUCAUCCAUCdTdT
hs-SNHG8-si-3	GGUGGUCCGUGAUAAUUAAdTdT	UUAAUUAUCACGGACCACCdTdT
NC	UUCUCCGAACGUGUCACGUdTdT	ACGUGACACGUUCGGAGAAdTdT
Fam NC	UUCUCCGAACGUGUCACGUdTdT	ACGUGACACGUUCGGAGAAdTdT
hs-TRPM7-si-1	CCACUGGACUUACGAAUAUTTdTdT	AUAUUCGUAAGUCCAGUGGTdTdT
hs-TRPM7-si-2	GGCACCUUUAUAUCAUUAATTdTdT	UUAAUGAUAUAAAGGUGCCTTdTdT
hs-TRPM7-si-3	GCAGCAGAGCCCGAUAUUATTdTdT	UAAUAUCGGGCUCUGCUGCTTdTdT

Supplemental Table 3. Primers used with qRT-PCR.

Species	Gene	Forward primer (5'-3')	Reverse primer (5'-3')
human	SNHG8	GCTGAGCTGAACACATTACG	ACGATAAGTCCATTGCCGGA
human	TRPM7	TGCCTCCTCCACTTATCATTCT	TACATTTCAACACACTGCTCTTCA
human	β-ACTIN	AAGAGCTACGAGCTGCCTGA	GGCAGTGATCTCCTTCTGCA

Supplemental Figures

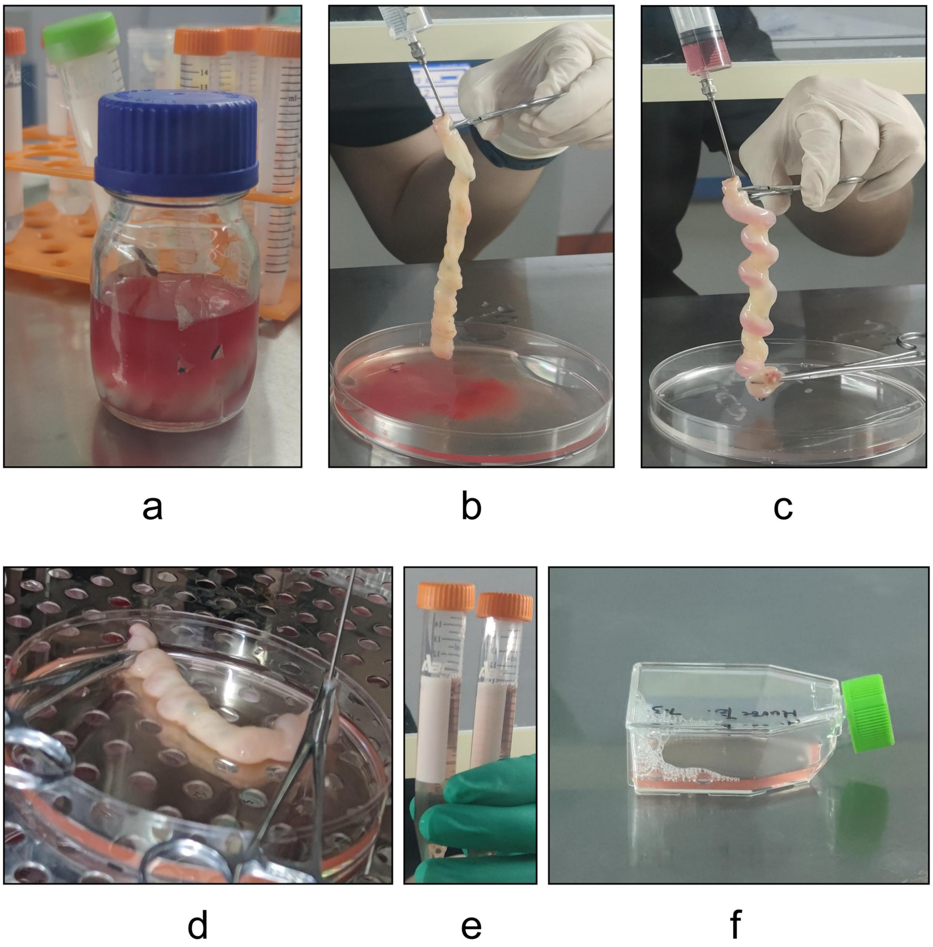

Figure S1. Flow chart of extraction of primary human umbilical vein endothelial cells（pHUVECs）. (a) the umbilical cord is preserved in culture medium. (b) the umbilical vein is washed with PBS. (c) 10ml of collagenase type I is perfused into the umbilical vein. (d) digested at 37°C for 20min. (e) collection of digestive fluid from umbilical veins. (f) after centrifugation, the ECM was resuspended and placed in a flask for culture.

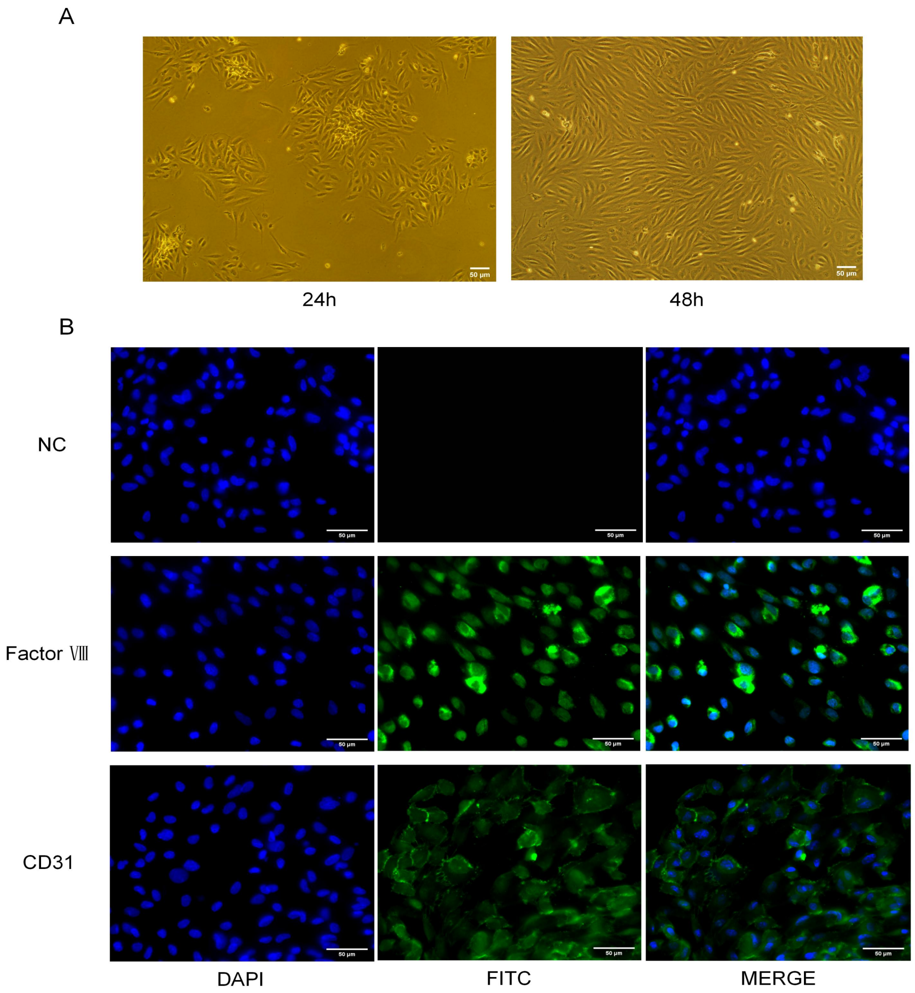

Figure S2. Identification of pHUVECs. (A) The growth situation of pHUVECs was observed at 24h and 48h by microscope (objective lens 100 times, scale=50uM); (B) Factor VIII and CD31 were detected by immunofluorescence method (note: blue: DAPI, green: FITC, objective lens 400 times, scale =50uM).

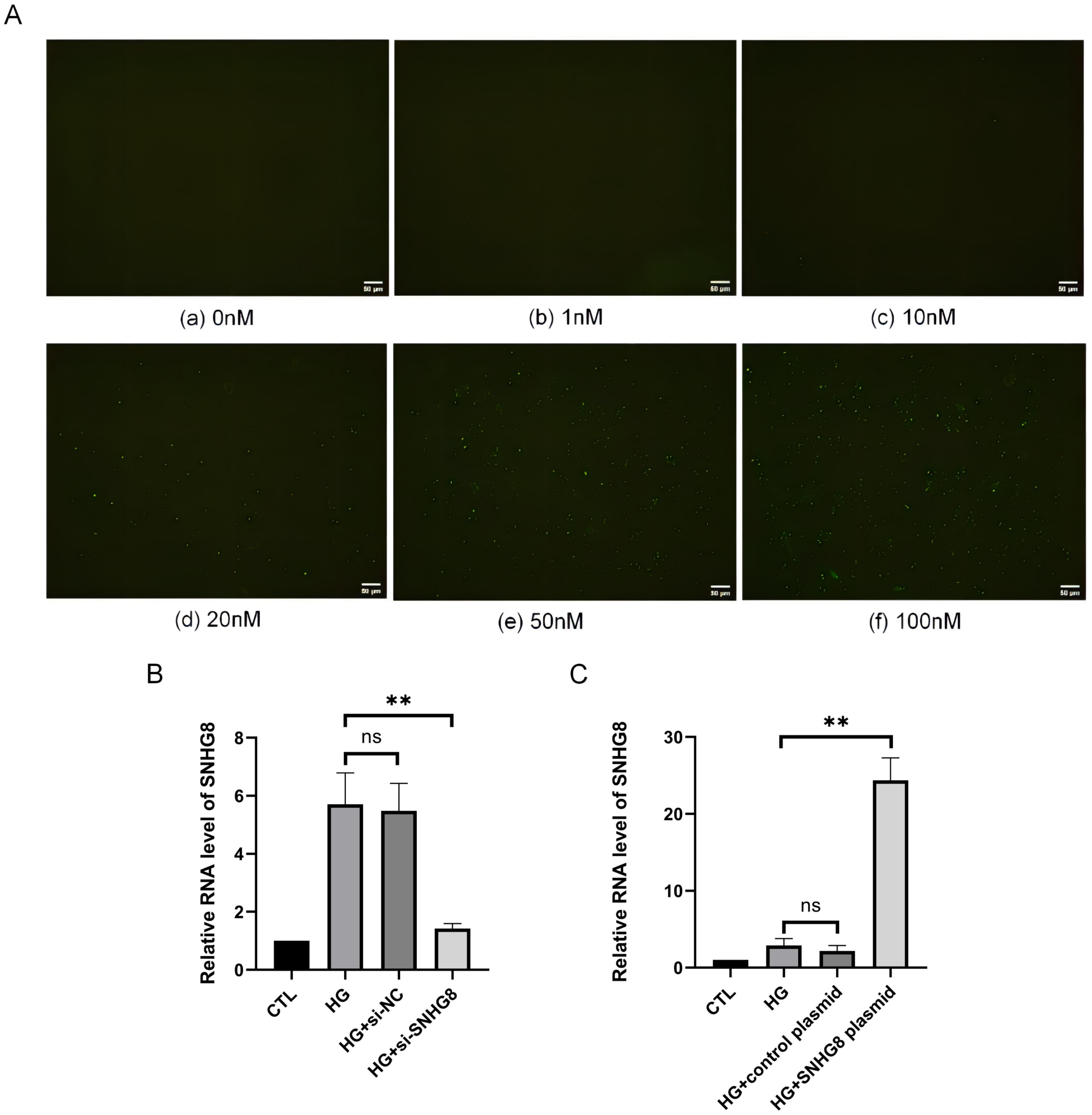

Figure S3. Effects of silencing and overexpression of SNHG8 on lncRNA SNHG8 expression in pHUVECs stimulated by high glucose. (A) Fluorescence microscopy was used to observe the fluorescence intensity of FAM-SiRNA transfected pHUVEC at different concentrations. a: blank control group (without siRNA, only RNAFIT); b: Transfection with 1nM siRNA+RNAFIT; c: Transfection with 10nM siRNA+RNAFIT; d: Transfection with 20nM siRNA+RNAFIT; d: Transfection with 50nM siRNA+RNAFIT; f: Transfection was performed with 100nM siRNA+RNAFIT; (B) After siRNA-SNHG8 was transfected into pHUVECs, pHUVECs was stimulated with HG (30mM) for 72 hours; (C) After SNHG8 plasmids were transfected into pHUVECs, pHUVECs was stimulated with HG (30mM) for 72 hours.The level of lncRNA SNHG8 was detected by RT-qPCR. (**,P<0.01;***,P<0.001,ns,P>0.05;n=3).

Original Images for Figures

Figure 2

Fig 2C

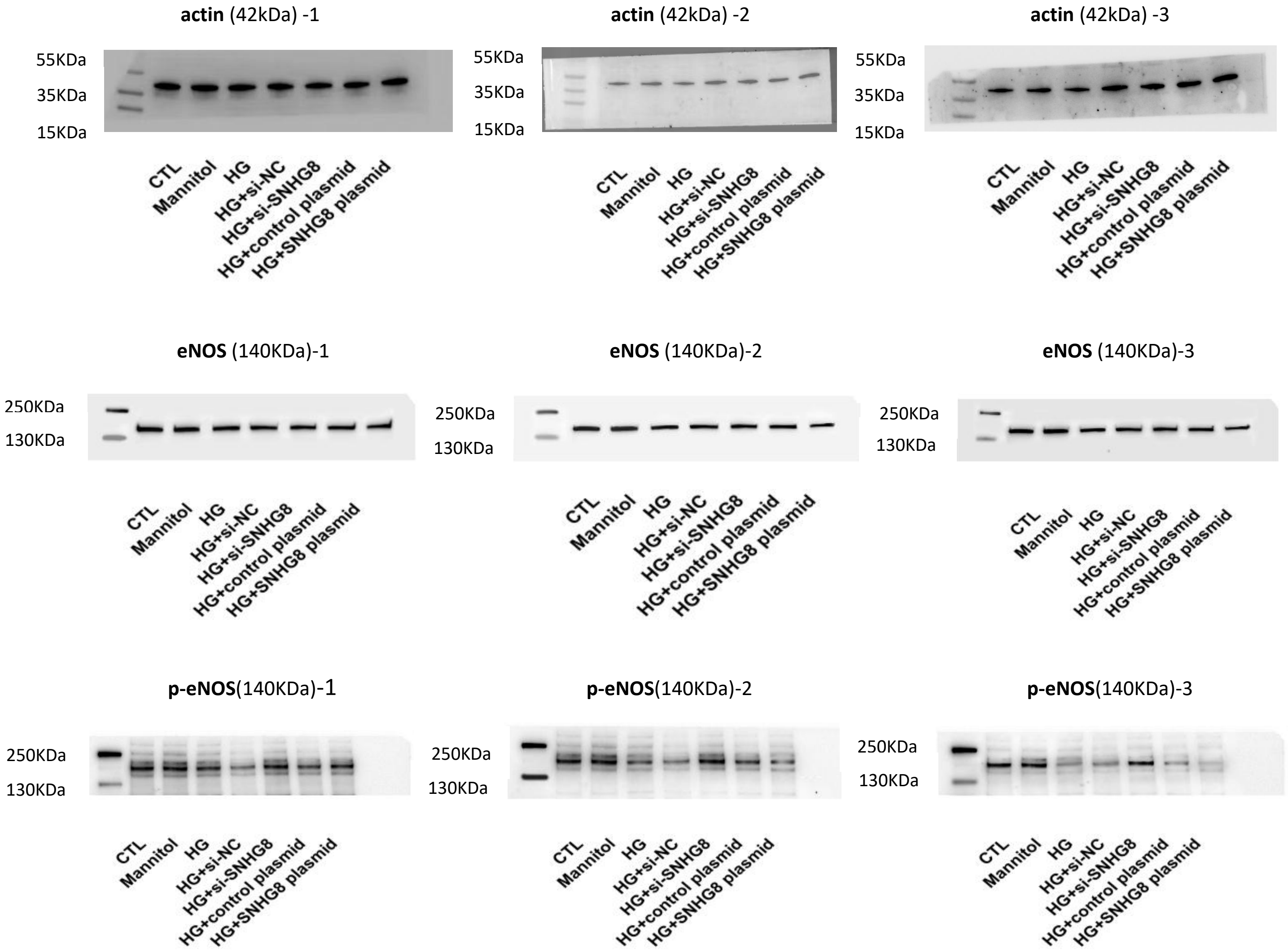

Marker:Thermo 26619(10-250KDa)

Fig 2E

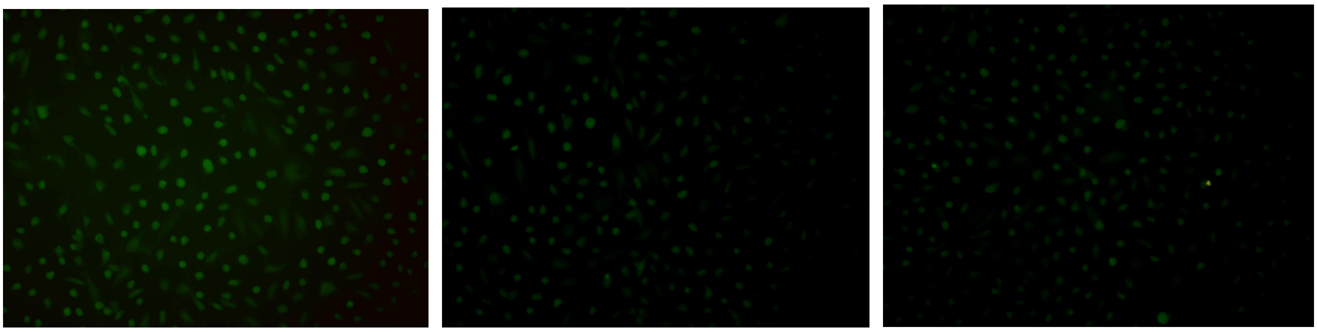

Fig 2E (CTL) Fig 2E (HG) Fig 2E (HG+control plasmid)

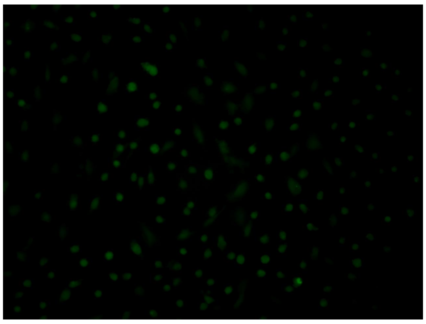

Fig 2E (HG+si-NC)

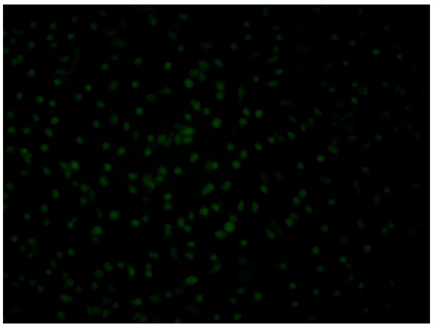

Fig 2E (HG+si-SNHG8)

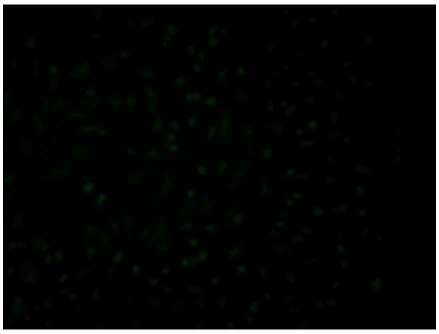

Fig 2E (HG+SNHG8 plasmid）

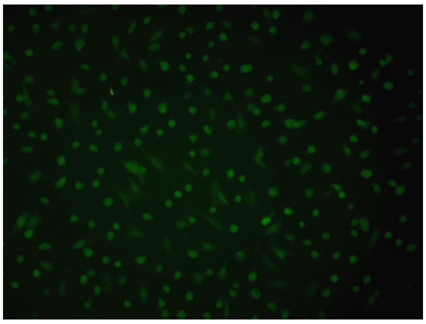

Fig 2E(Mannitol)

Figure 3

Fig 3A

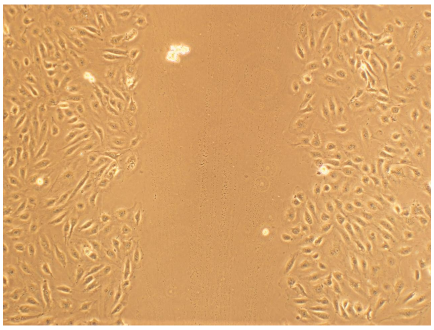

Fig 3A (CTL-0hour)

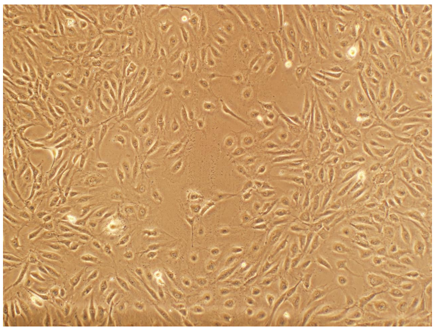

Fig 3A (CTL-24hours)

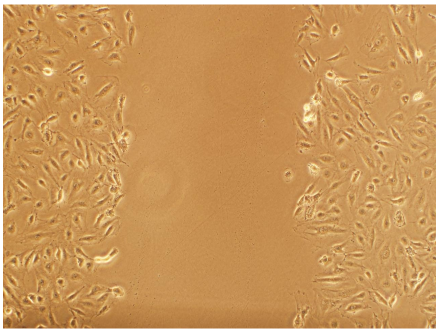

Fig 3A (HG+control plasmid-0hour)

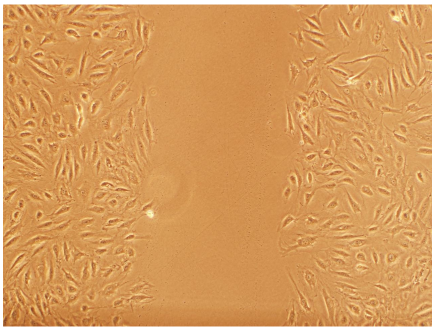

Fig 3A (HG+control plasmid-24hours)

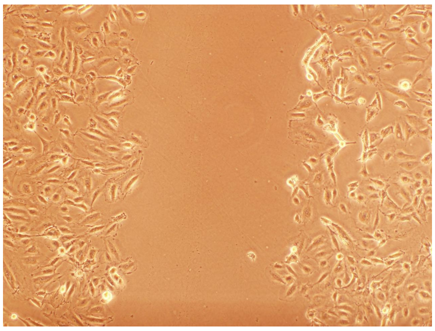

Fig 3A (HG+si-NC-0hour)

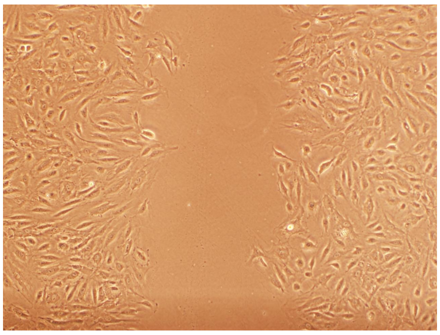

Fig 3A (HG+si-NC-24hours)

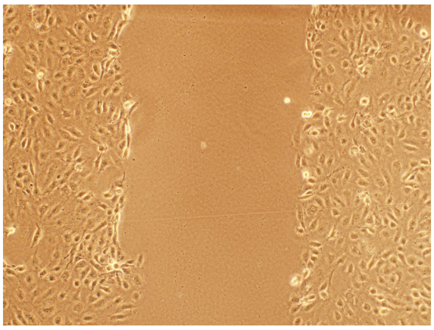

Fig 3A (HG+si-SNHG8-0hour)

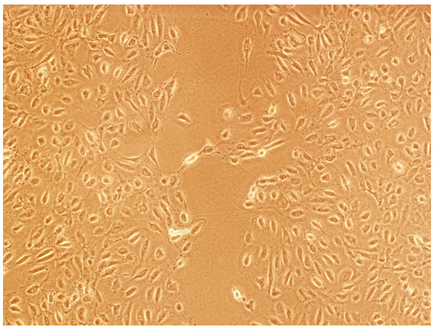

Fig 3A (HG+si-SNHG8-24hours)

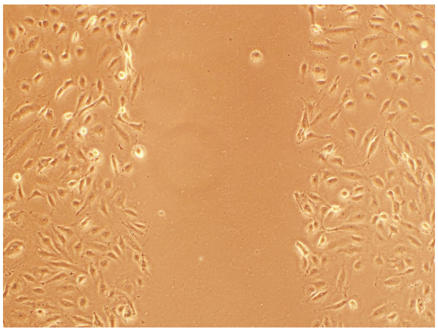

Fig 3A (HG+SNHG8 plasmid-0hour)

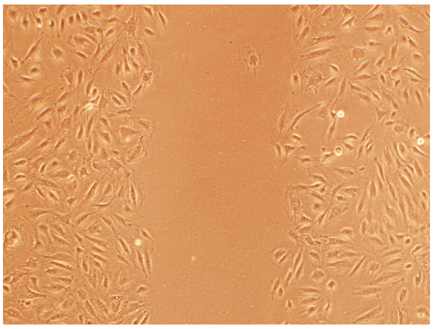

Fig 3A (HG+SNHG8 plasmid-24hours)

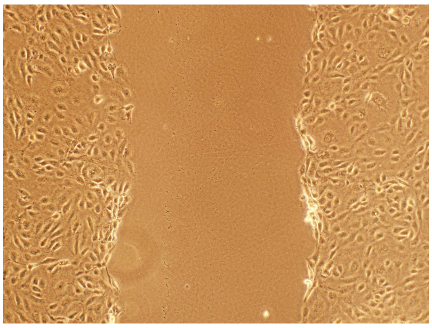

Fig 3A (HG-0hour)

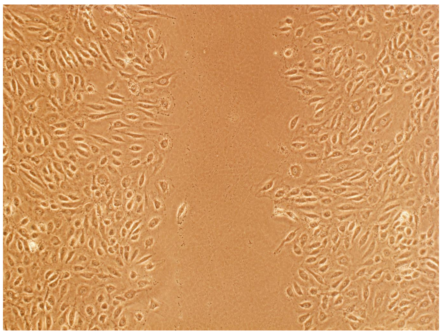

Fig 3A (HG-24hours)

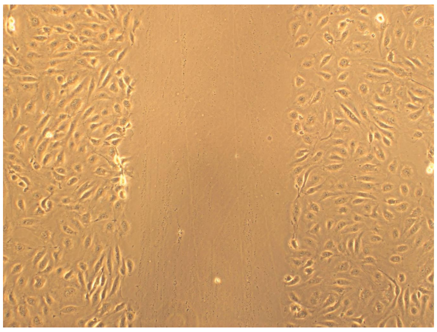

Fig 3A (Mannitol-0hour)

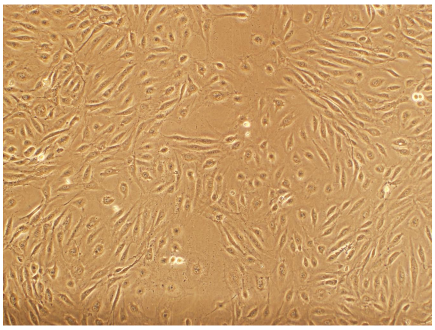

Fig 3A (Mannitol-24hours)

Fig 3 C

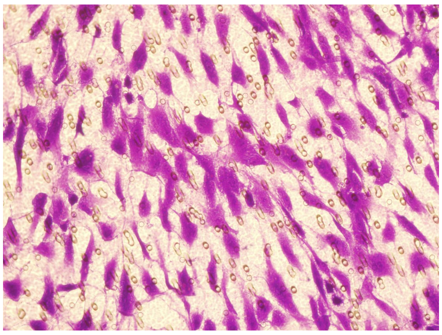

Fig 3 C(CTL)

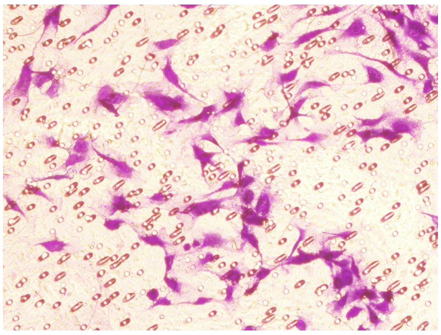

Fig 3 C(HG)

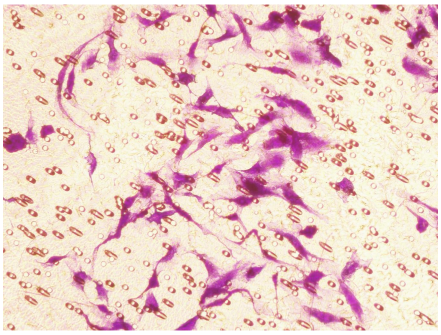

Fig 3 C(HG+control plasmid)

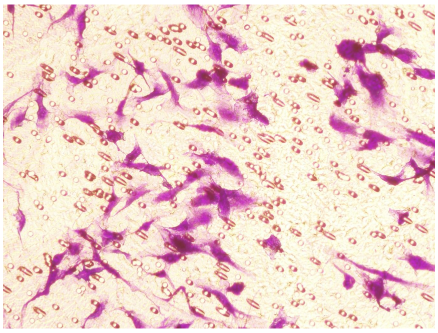

Fig 3 C(HG+si-NC)

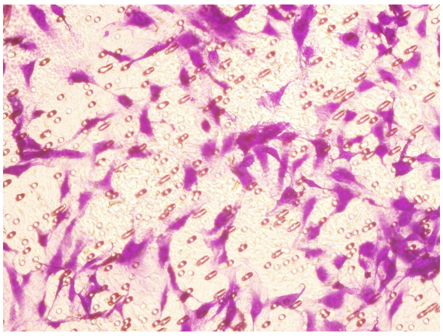

Fig 3 C(HG+si-SNHG8)

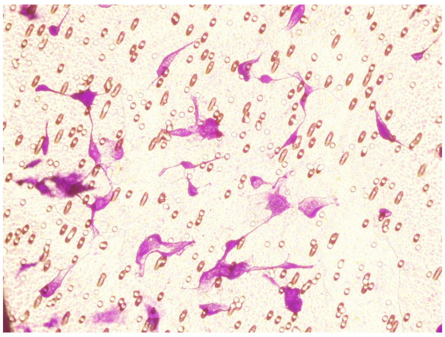

Fig 3 C(HG+SNHG8 plasmid)

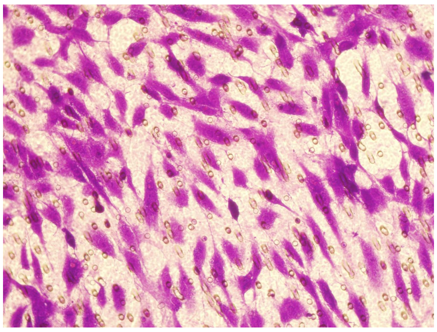

Fig 3 C(Mannitol)

Fig 3E

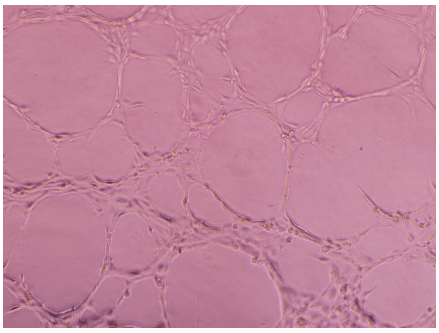

Fig 3E(CTL)

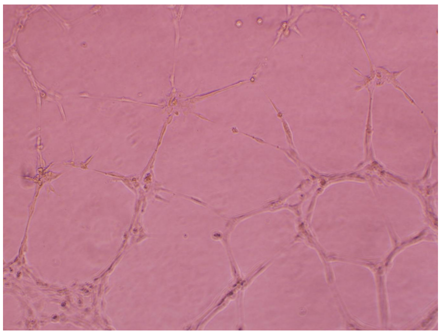

Fig 3E(HG)

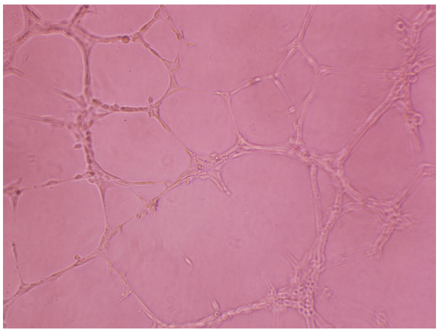

Fig 3E(HG+control plasmid)

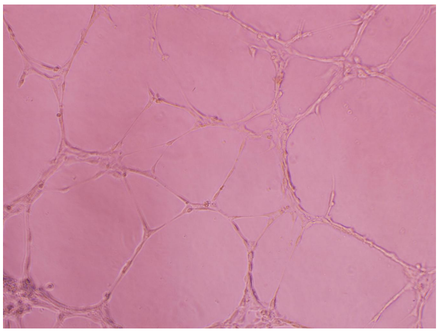

Fig 3E(HG+si-NC)

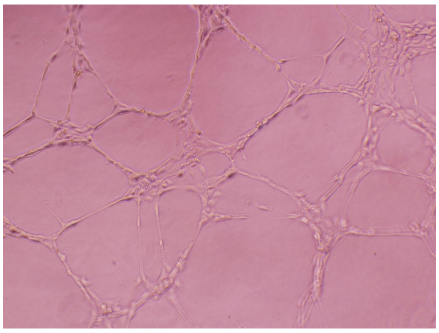

Fig 3E(HG+si-SNHG8)

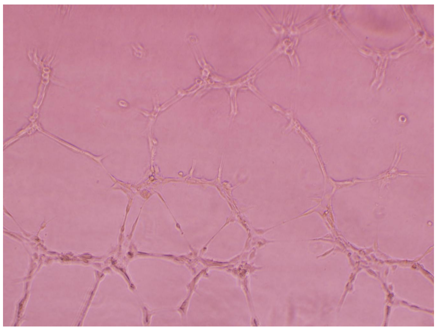

Fig 3E(HG+SNHG8 plasmid)

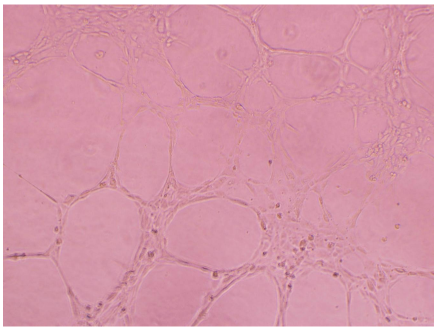

Fig 3E(Mannitol)

Figure 4

Fig 4A

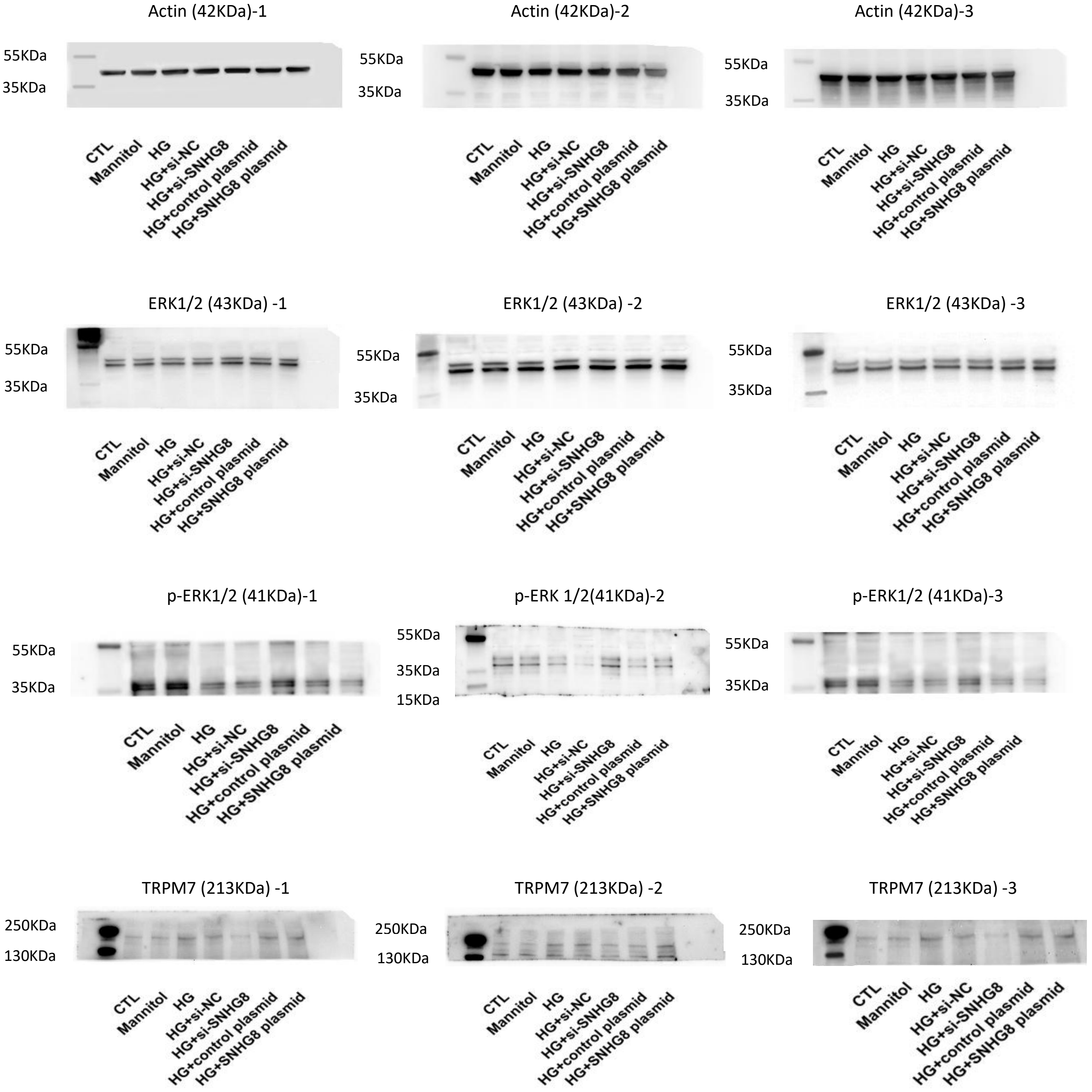

Marker:Thermo 26619(10-250KDa)

Fig 4D

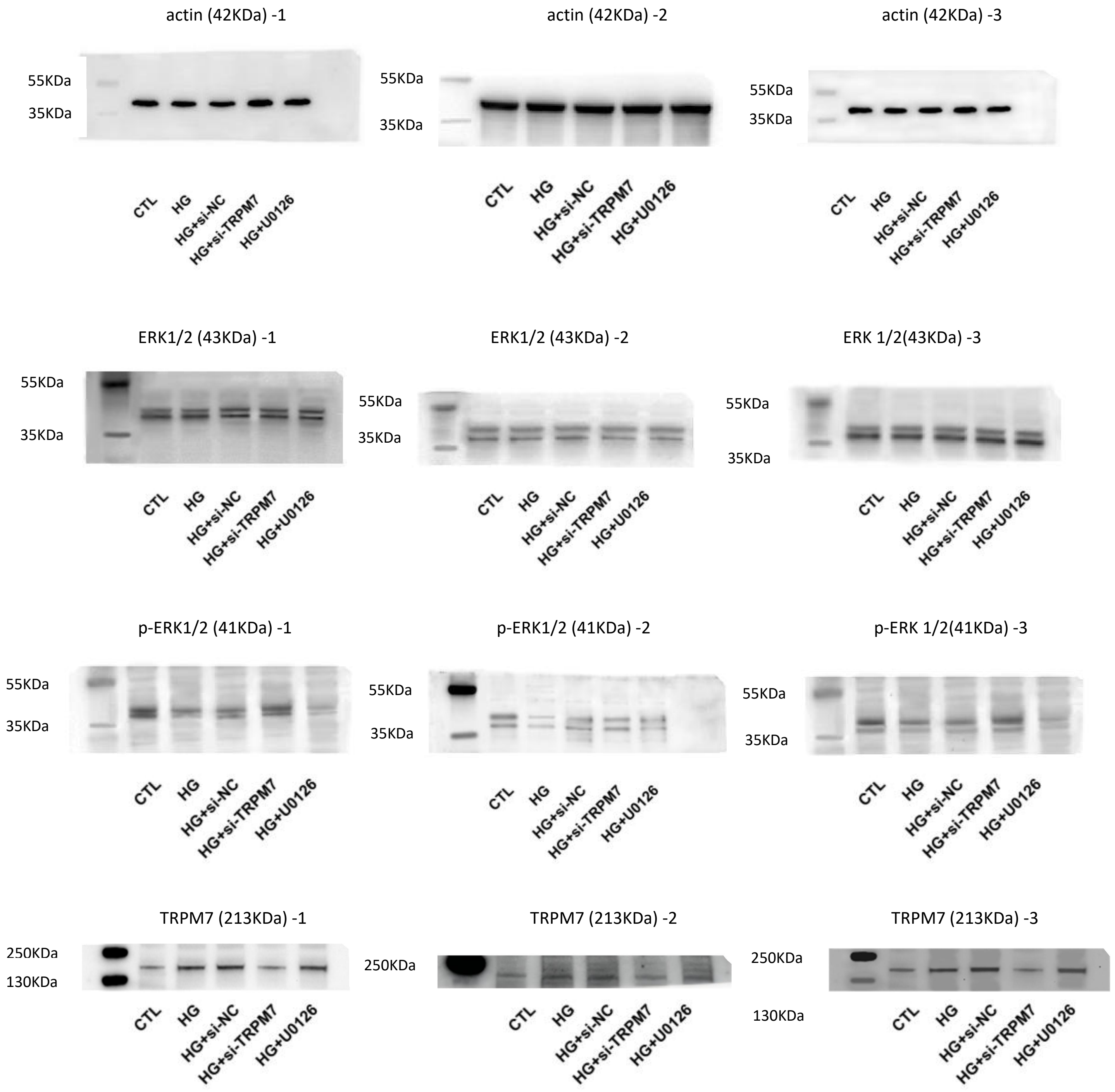

Marker:Thermo 26619(10-250KDa)

Figure 5

Fig 5A

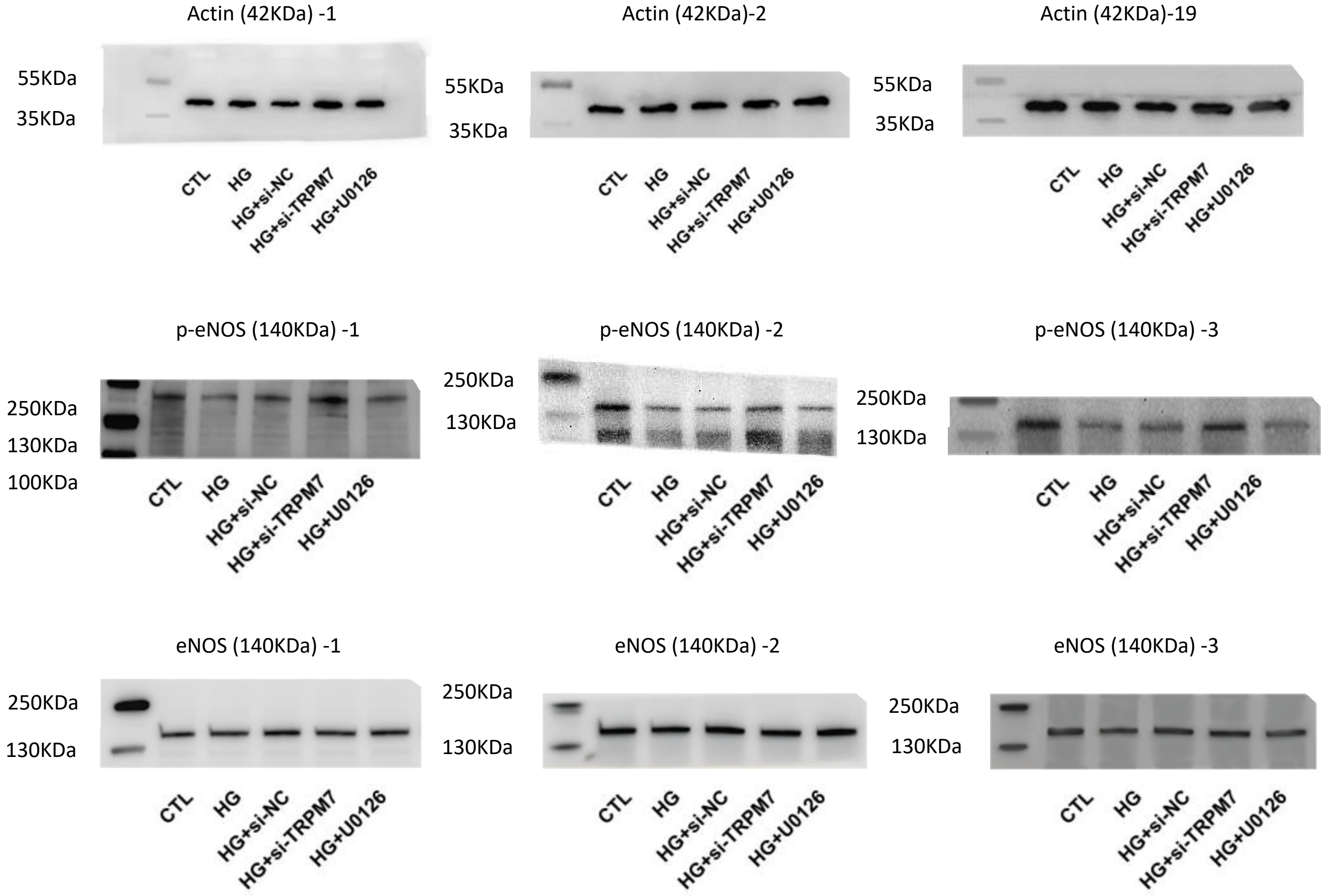

Marker:Thermo 26619(10-250KDa)

Fig 5C

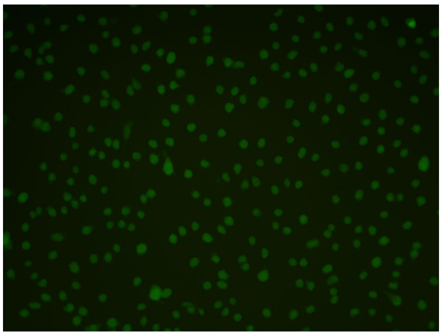

Fig 5C (CTL)

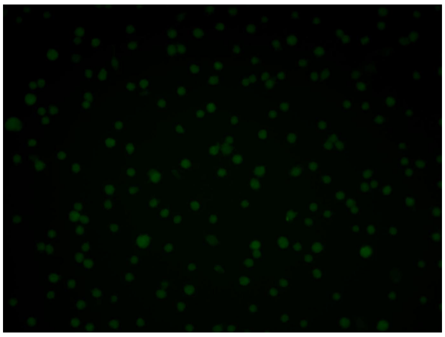

Fig 5C (HG)

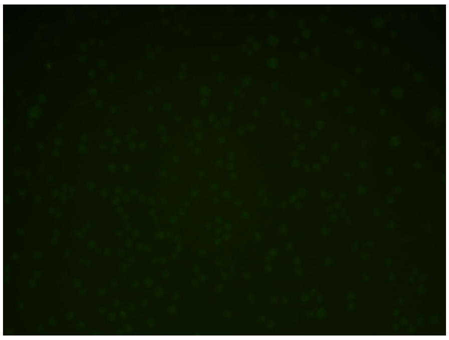

Fig 5C (HG+si-NC)

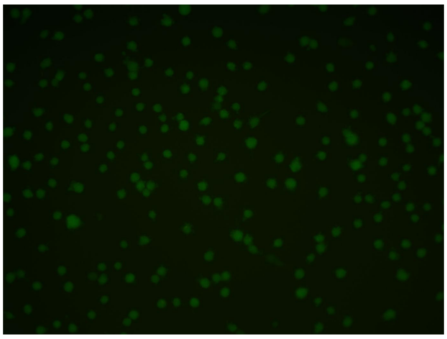

Fig 5C (HG+si-TRPM7)

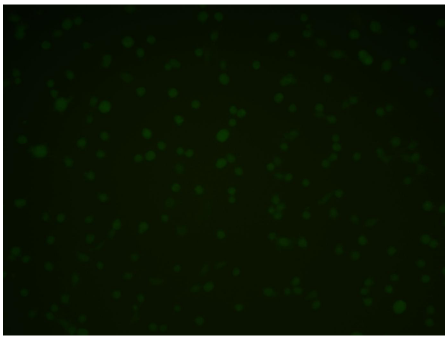

Fig 5C (HG+U0126)

Figure 6

Fig 6A

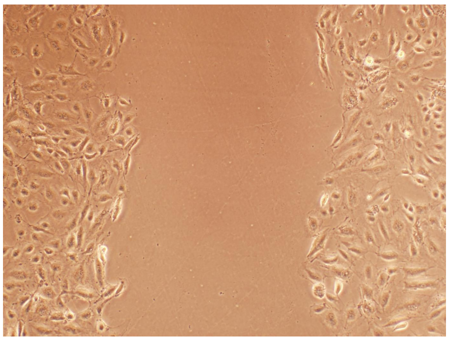

Fig 6A (CTL-0hour)

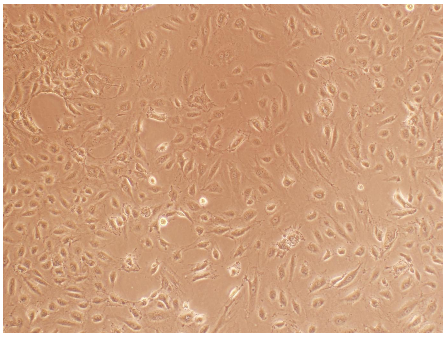

Fig 6A (CTL-24hours)

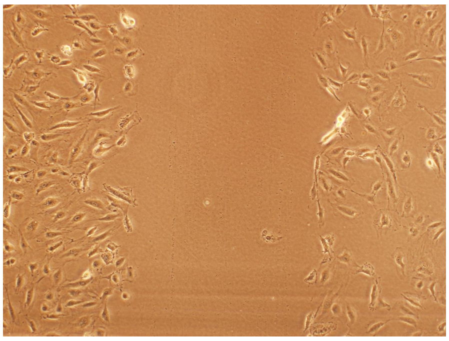

Fig 6A (HG+si-NC-0hour)

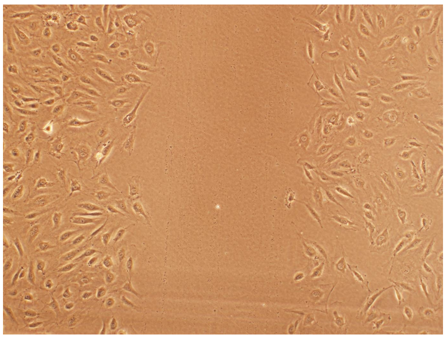

Fig 6A (HG+si-NC-24hours)

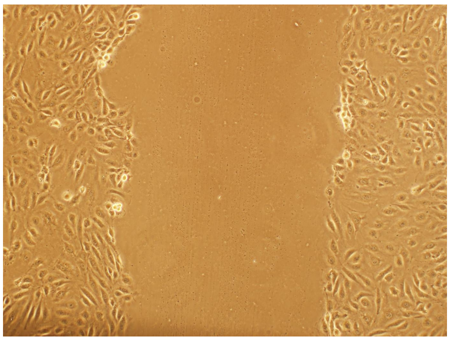

Fig 6A (HG+si-TRPM7-0hour)

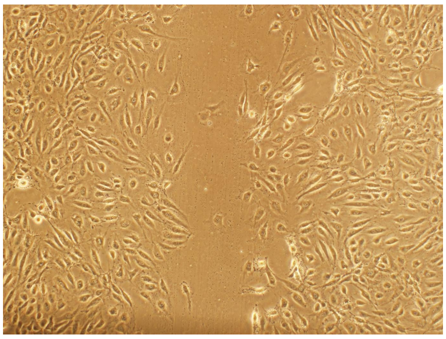

Fig 6A (HG+si-TRPM7-24hours)

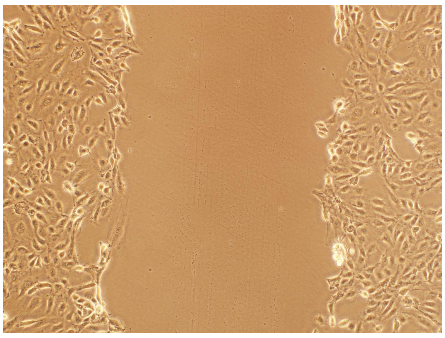

Fig 6A (HG+U0126-0hour)

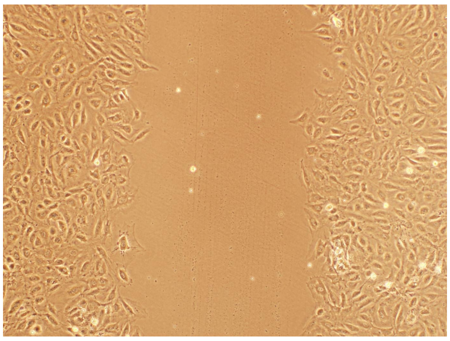

Fig 6A (HG+U0126-24hours)

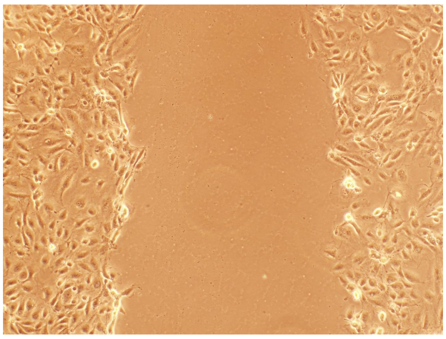

Fig 6A (HG-0hour)

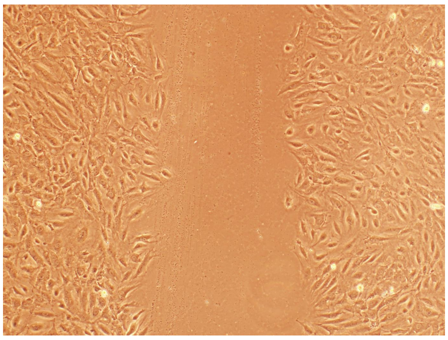

Fig 6A (HG-24hours)

Fig 6C

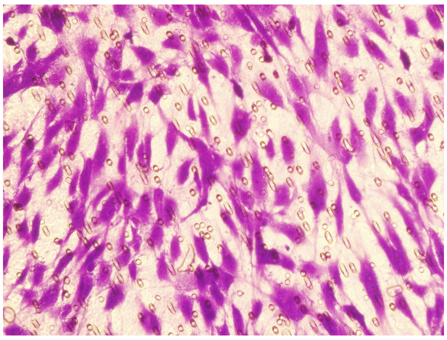

Fig 6C (CTL)

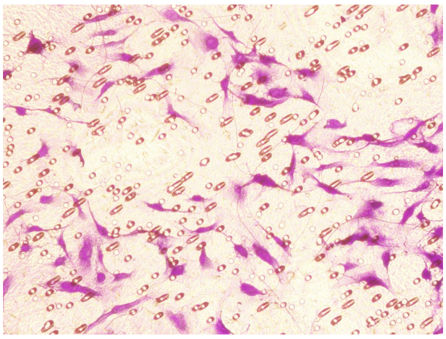

Fig 6C (HG)

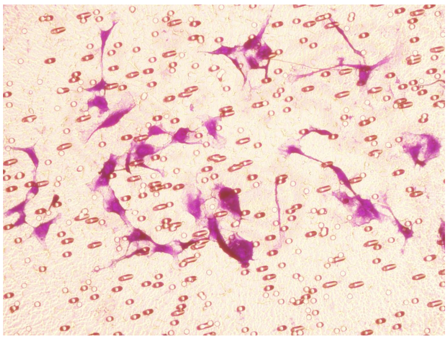

Fig 6C (HG+si-NC)

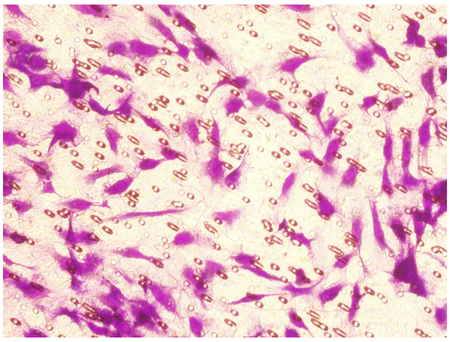

Fig 6C (HG+si-TRPM7)

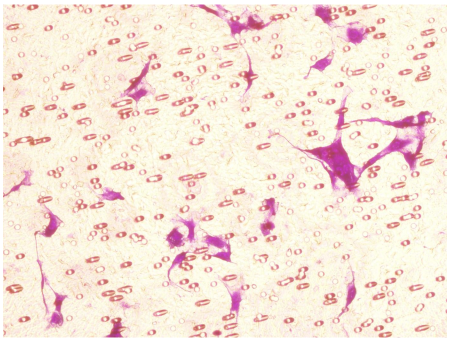

Fig 6C (HG+U0126)

Fig 6E

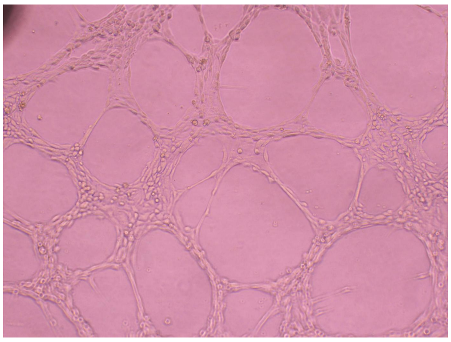

Fig 6E(CTL)

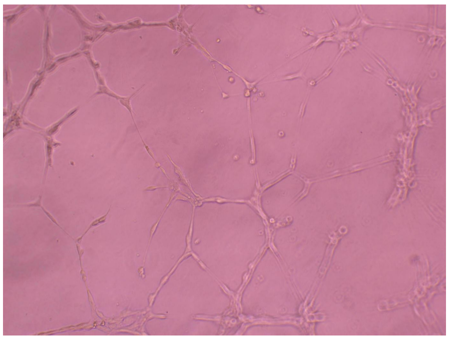

Fig 6E(HG)

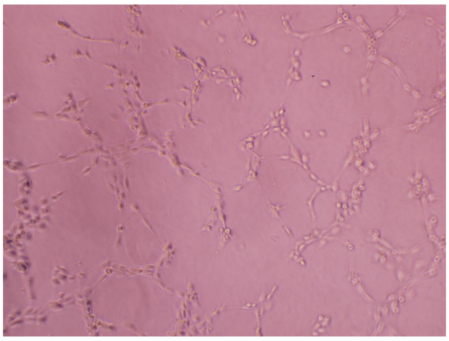

Fig 6E(HG+si-NC)

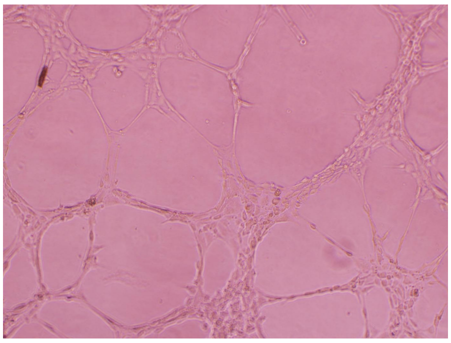

Fig 6E(HG+si-TRPM7)

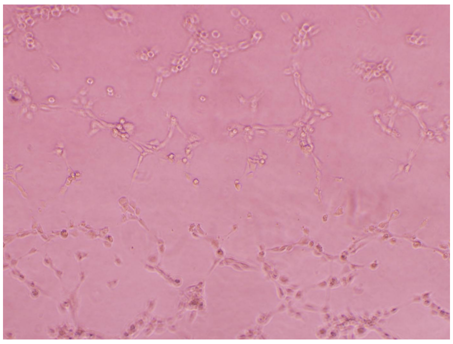

Fig 6E(HG+U0126)
